# An fMRI study of crossmodal emotional congruency and the role of semantic content in the aesthetic appreciation of naturalistic art

**DOI:** 10.3389/fnins.2025.1516070

**Published:** 2025-07-30

**Authors:** Funda Yilmaz, Tessa M. van Leeuwen, Umut Güçlü, Yağmur Güçlütürk, Rob van Lier

**Affiliations:** ^1^Donders Institute for Brain, Cognition and Behaviour, Radboud University, Nijmegen, Netherlands; ^2^Department of Communication and Cognition, Tilburg School of Humanities and Digital Sciences, Tilburg University, Tilburg, Netherlands

**Keywords:** audiovisual, emotion, art, aesthetics, beauty ratings, fMRI, multisensory integration, semantics

## Abstract

Numerous studies have explored crossmodal correspondences, yet have so far lacked insight into how crossmodal correspondences influence audiovisual emotional integration and aesthetic beauty. Our study investigated the behavioral and neural underpinnings of audiovisual emotional congruency in art perception. Participants viewed ‘happy’ or ‘sad’ paintings in an unimodal (visual) condition or paired with congruent or incongruent music (crossmodal condition). In the crossmodal condition, the music could be emotionally congruent (e.g., happy painting, happy music) or incongruent with the painting (e.g., happy painting, sad music). We also created Fourier Scrambled versions of each painting to test for the influence of semantics. We tested 21 participants with fMRI while they rated the presentations. Beauty ratings did not differ for unimodal and crossmodal presentations (when aggregating across incongruent and congruent crossmodal presentations). We found that crossmodal conditions activated sensory and emotion-processing areas. When zooming in on the crossmodal conditions, the results revealed that emotional congruency between the visual and auditory information resulted in higher beauty ratings than incongruent pairs. Furthermore, semantic information enhanced beauty ratings in congruent trials, which elicited distinct activations in related sensory areas, emotion-processing areas, and frontal areas for cognitive processing. The significant interaction effect for Congruency × Semantics, controlling for low-level features like color and brightness, observed in the behavioral results was further revealed in the fMRI findings, which showed heightened activation in the ventral stream and emotion-related areas for the congruent conditions. This demonstrates that emotional congruency not only increased beauty ratings but also increased the in-depth processing of the paintings. For incongruent versus congruent comparisons, the results suggest that a frontoparietal network and caudate may be involved in emotional incongruency. Our study reveals specific neural mechanisms, like ventral stream activation, that connect emotional congruency with aesthetic judgments in crossmodal experiences. This study contributes to the fields of art perception, neuroaesthetics, and audiovisual affective integration by using naturalistic art stimuli in combination with behavioral and fMRI analyses.

## 1 Introduction

Our understanding of the world relies on interpreting environmental cues we receive from continuous sensory input, particularly from visual and auditory information (Vuilleumier, 2005). In contrast to pure multisensory audiovisual integration, crossmodal correspondence is the phenomenon in which features from different sensory modalities naturally align and associate (Spence, 2011). The underlying mechanisms of crossmodal correspondences have been widely investigated (e.g., Giannos et al., 2021; Mok et al., 2019; Motoki et al., 2020; Saluja and Stevenson, 2018) by using pairs of different sensory modalities, including, e.g., color and music (Palmer et al., 2013), taste and music (Wang et al., 2016) and odors and music (Levitan et al., 2015). In a review by Spence (2011), the mechanisms underlying crossmodal correspondences are categorized as structural, statistical, emotional, or semantic correspondences. Spence (2011) also highlights that emotional crossmodal correspondences connect sensations that evoke similar emotions; for example, happiness can be associated with both the color yellow and major musical tones. On the other hand, semantic correspondences are based on congruent semantic relationships, for example, pairing a meowing sound with a static image of a cat as congruent or with a dog as incongruent (Spence, 2011; Hein et al., 2007; Molholm et al., 2004; Spence and Deroy, 2013).

There is substantial evidence that emotional correspondences significantly contribute to high-level multisensory integration (e.g., Pelowski, 2017; Spence, 2020; Wang et al., 2017). An important factor in this process is how emotional (in)congruence influences the overall affective state. Studies suggest that congruent emotions coming from auditory and visual domains facilitate emotional experience (Baumgartner et al., 2006; Christensen et al., 2014; Gao et al., 2018; Gao et al., 2019; Rosenfeld and Steffens, 2019). Imagine watching a movie where a key scene shows the main character’s tragic death. You would expect the background music to be slow and sorrowful, congruent with the emotion. Similarly, in a joyful wedding scene with everyone smiling, upbeat and happy music seems fitting. According to Spence, crossmodal correspondences between complex auditory and visual stimuli significantly influence our emotional responses through emotional correspondences (Spence, 2020). Building on this premise, our study examines unimodal (paintings) effects, crossmodal (music and paintings) emotional (in)congruency, and its influence on aesthetic experience, reflecting growing interest in crossmodal aesthetic interactions.

Several studies have compared congruent crossmodal (audiovisual) stimuli with unimodal auditory or visual presentations (Kreifelts et al., 2007, 2010; Robins et al., 2009). Some studies focus on emotion perception, particularly on recognizing social emotion cues, showing that matching facial expressions with the tone of voice or emotional prosody enhances emotion recognition (De Gelder and Vroomen, 2000). Kreifelts et al. (2007) demonstrated that subjective ratings for the crossmodal presentation of congruent face and voice expressions significantly increased emotional experience compared to unimodal presentations (pictures alone). While comparisons of crossmodal and unimodal presentations have thus been explored, recent research has increasingly focused on understanding the intricacies of crossmodal interactions and on the role of emotional (in)congruency (Dolan et al., 2001; Müller et al., 2011; Gao et al., 2018; Gao et al., 2020; Christensen et al., 2014; Baumgartner et al., 2006).

The congruency of emotional information from auditory (music) and visual (faces) sources has been examined across behavioral and neuroimaging studies. For instance, Jeong et al. (2011) found in the behavioral ratings that the congruency of the music influenced the emotional ratings of faces: happy music increased ratings of happiness in happy faces and decreased sadness in sad faces (Jeong et al., 2011). Studies using functional magnetic resonance imaging (fMRI) demonstrated that emotional congruency enhanced activity across various brain regions, including the superior temporal cortex, amygdala, posterior/middle cingulate cortex, superior frontal cortex, insula, thalamus (Jansma et al., 2014; Klasen et al., 2011; Müller et al., 2011; Dolan et al., 2001; Petrini et al., 2011). For example, Klasen et al. (2011) studied emotional faces and voices in congruent or incongruent conditions using fMRI during an emotional classification task. The authors found that congruent emotions activated the amygdala, insula, ventral posterior cingulate, temporo-occipital, and auditory cortices. In contrast, incongruent emotions triggered a frontoparietal network and the bilateral caudate nucleus, suggesting increased processing demands on working memory and emotion-encoding regions. Gao et al. (2020) studied brain responses to audiovisual valence congruency, pairing positive or negative video clips with matching music. Their analysis revealed distinct neural patterns in areas such as the bilateral superior temporal cortex and right anterior cingulate, differentiating congruent from incongruent emotional valence. The majority of these studies have either focused on discrete emotions by using face-voice pairs (Dolan et al., 2001; Müller et al., 2011) or concentrated on emotional valence (Gao et al., 2018; Gao et al., 2019; Christensen et al., 2014; Baumgartner et al., 2006). While these fMRI studies provide insights into emotional congruency, the neural basis of emotional (in)congruency with naturalistic stimuli remains underexplored.

While previous research has primarily examined congruency in audiovisual pairings, often focusing on artistic style or complexity between music and paintings (Albertazzi et al., 2020; Isaacson et al., 2023), the role of emotional (in)congruency—particularly the happy-sad pairing—and its influence on aesthetic judgments has been less explored. Additionally, art has long been recognized for its ability to evoke strong emotions, with aesthetic theories acknowledging its integral role in shaping emotional experiences (Tan, 2000; Silvia, 2005). However, there has been limited exploration of how emotional congruency influences aesthetic experiences, particularly when using artistic stimuli in crossmodal contexts. Differing from earlier audiovisual affective studies, our research uses artistic stimuli—music and paintings—and investigates how emotional congruency and incongruency affect beauty ratings within crossmodal experiences.

We explore how the emotional congruency between visual (paintings) and auditory stimuli (music) influences the perceived beauty of paintings and investigate the underlying neural mechanisms by comparing crossmodal and unimodal presentations. We hypothesize that paintings in congruent trials will receive higher beauty ratings than those in incongruent trials (Van Lier and Koning, 2017), and for the fMRI results, we predict greater activation in audiovisual integration and emotion processing areas during crossmodal trials, in contrast to visual areas engaged by unimodal trials. Emotional congruency is also hypothesized to trigger greater activations in areas associated with audiovisual integration and emotion processing, compared to emotional incongruency in line with research showing activations in the temporo-occipital cortex, amygdala, and insula (Klasen et al., 2011; Gao et al., 2020). Overall, this study aims to shed light on the neural mechanisms underlying the interaction between emotional congruency and the aesthetic experience of art.

We presented paintings and musical excerpts with happy/sad valences (see also Van Lier and Koning, 2017). In the crossmodal condition, the music could be emotionally congruent (e.g., happy painting, happy music) or incongruent with the painting (e.g., happy painting, sad music). Importantly, we selected specific emotions (happy versus sad), which are known to be easily applied to both visual and auditory stimuli (Augustin et al., 2012). Moreover, we aimed to disentangle the effects of the specific colors and the effects of the semantics of the visual scene artwork. For example, a sad scene (depicting somebody dying) may be depicted in darker colors (e.g., brown, purple, grey), whereas happy scenes may be depicted in brighter colors (e.g., yellow, light blue). Furthermore, the study by Palmer et al. (2013) showed that music-color associations are strongly mediated by emotional content, with faster, major-mode music eliciting brighter, more saturated color choices and slower, minor-mode music evoking darker, desaturated colors. In that case, both the colors and the semantics of the scene may contribute to the congruence with the music. To account for that, we incorporated Fourier Scrambled versions of each painting (Wintermans, 2019), in which the semantic information was lost. Therefore, our selected stimuli set, evoking distinct emotional responses, allows us to investigate how the brain integrates these affective crossmodal experiences.

## 2 Methods

Before the current study, a behavioral pre-experiment was conducted to select stimuli carefully (Wintermans, 2019). In this initial phase, participants rated various paintings and music excerpts on perceived happiness and sadness, allowing us to identify stimuli that elicited robust emotional responses. With these selected stimuli, we performed an fMRI experiment, while behavioral data were additionally collected within the scanner.

### 2.1 Stimulus selection pre-experiment

**Figure 1:**
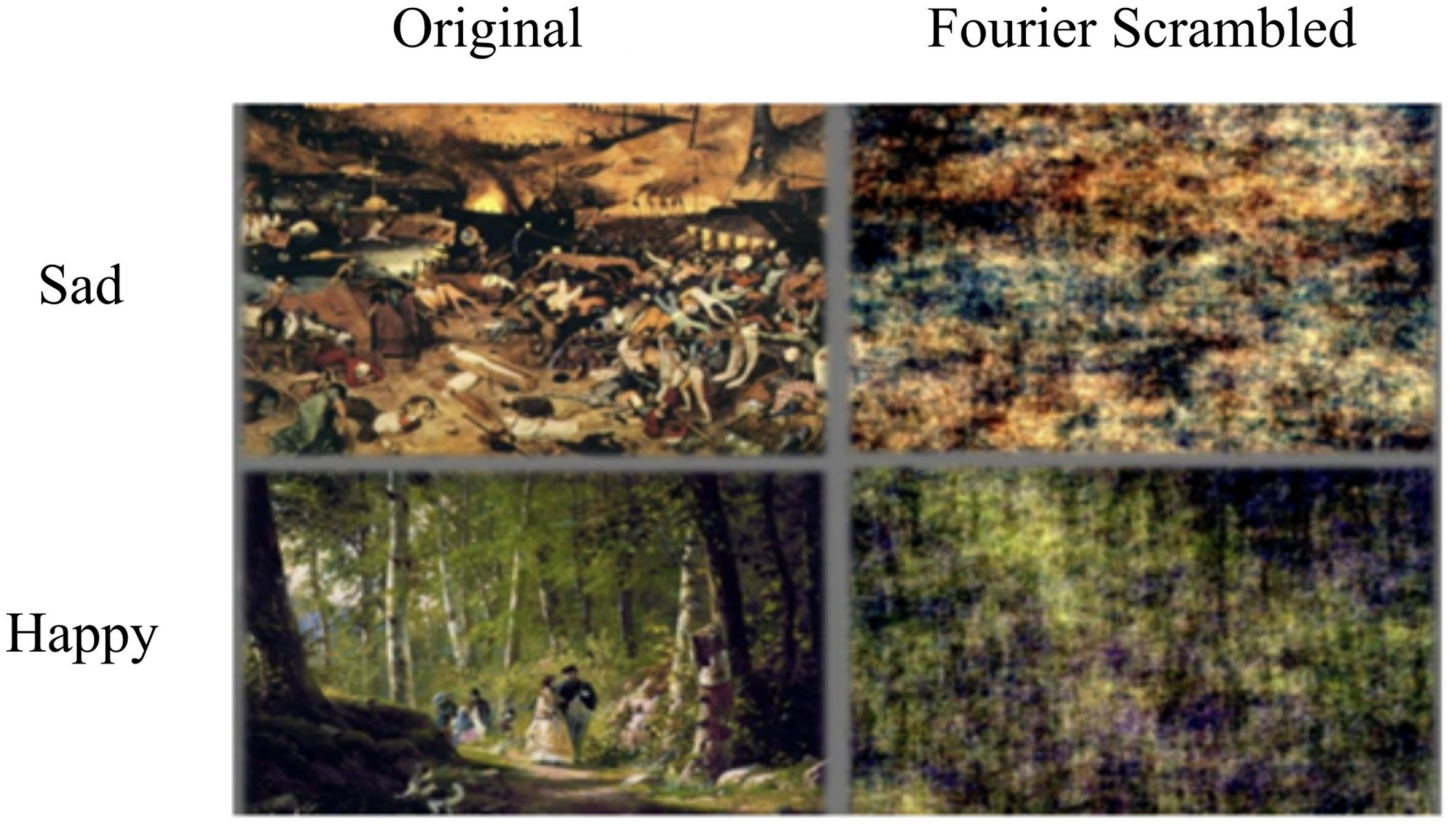
Example stimuli. A sad and happy painting in their original and Fourier Scrambled version.

Stimuli for the pre-experiment were obtained from the freely available Art UK (Public Catalogue Foundation, n.d.), WikiArt databases for paintings (WikiArt, n.d.), and the MagnaTagATune database (Law et al., 2007; Law and Von Ahn, 2009) for music. The stimuli were initially chosen based on already available tags indicating happiness and sadness. Details of the stimulus selection process are described in the report of the pre-experiment (Wintermans, 2019); here, we briefly summarize the main steps. The figurative paintings contained semantic information that evoked emotions (happy/sad), while the music excerpts were purely instrumental. Participants in the selection experiment rated the elicited emotion on a 9-point rating scale, going from “extremely sad” to “extremely happy.” For the fMRI experiment, we selected the 20 paintings that received the highest ratings for either happiness or sadness. Examples of sad and happy paintings for both the original and Fourier Scrambled versions are shown in Figure 1.

During stimulus selection, we manipulated paintings by applying Fourier scrambling to evaluate the impact of the manipulation on emotional ratings. The Fourier Scrambled versions were generated by adding a random phase structure to the original phase spectrum of the images, combining it with the amplitude spectrum, and performing an inverse Fourier transform in MATLAB (The MathWorks Inc., 2021). Both original and Fourier Scrambled versions of these paintings were used, while music excerpts were retained in their original form. The chosen paintings were modified to achieve a square format and resized to dimensions of 600 × 600 pixels. Similarly, music excerpts were trimmed to 10 s, ensuring uniformity in the duration and size of all visual and auditory stimuli (Wintermans, 2019). These trimmed excerpts were rated for their emotional valence (happy-sad dimension) in this behavioral experiment. The selected stimuli, both visual and auditory, were utilized in the subsequent fMRI experiment.

### 2.2 Stimuli—fMRI experiment

The fMRI study had a factorial design comprising two main factors: Modality—Unimodal (only visual painting stimuli) versus Crossmodal (the visual paintings together with auditory music stimuli), and Semantics—Original versus Fourier Scrambled paintings, to control for the influence of semantic information from the paintings. Next, another manipulation was added related to emotional congruence/incongruence (combining music with visual paintings). Each painting and musical excerpt has an emotional valence, either tagged as happy or sad, based on the behavioral pre-experiment. Therefore, within the Crossmodal factor, we have two levels of Emotional Congruency: either emotionally congruent across visual and auditory domains or emotionally incongruent. The trial types are summarized in Tables 1, 2. Additionally, we performed a color analysis of original paintings, comparing the brightness of happy and sad paintings. We computed brightness using custom Python code by converting images to 8-bit grayscale, where each pixel has a single intensity value from 0 (black) to 255 (white). The average of these values gives a scalar measure of image brightness. The results showed that happy paintings were significantly brighter (mean brightness: 163.08) compared to sad paintings (mean brightness: 89.38). The Fourier Scrambled versions of the paintings maintained these low-level differences in happy/sad valences while removing differences related to semantic content. We used 20 unique paintings (10 happy, 10 sad) along with 20 Fourier Scrambled versions of these paintings. Furthermore, we incorporated 20 unique music excerpts (10 happy, 10 sad) to complement the visual stimuli.

**Table 1:** Trial types for the factors modality and semantics (containing all trials).

Modality	Semantics	Trial number
Unimodal	Original	80
	Fourier Scrambled	80
Crossmodal	Original	160
	Fourier Scrambled	160

**Table 2:** Trial types for the factors of emotional congruency and semantics within the crossmodal trials.

Emotional congruency	Semantics	Trial number
Crossmodal - emotionally congruent (happy painting/happy music or sad painting/sad music)	Original	80
	Fourier Scrambled	80
Crossmodal - emotionally incongruent (happy painting/sad music or sad painting/happy music)	Original	80
	Fourier Scrambled	80

### 2.3 Experimental design and procedure

Stimulus presentation in the fMRI scanner was conducted using PsychoPy (Peirce, 2007, 2009) on a 32-inch BOLDscreen (Cambridge Research). Participants viewed the screen through a visual surface mirror attached to the head coil, allowing them to see the stimuli.

The experiment was divided into two scanning sessions, each consisting of four runs. Each run included 60 trials per run and 480 trials for the entire experiment across two sessions (240 trials per session) (see Tables 1, 2). The trials within each run were presented in a random order and only demonstrated once throughout the experiment. To minimize the potential effects of recognition, parallel trials of original paintings and their Fourier Scrambled versions were not presented in the same scanning session. Original and Fourier Scrambled trials were presented within each run and across both sessions on the same day rather than on separate days.

The experiment began with on-screen instructions and was preceded by five practice trials, during which no scanning took place. In the main experiment, before the start of each trial, there was a blank screen for 0.75 s plus a variable jitter period (pre-stimulus interval). The jitter duration varied between zero, one, or two times the TR (1.5 s). After this blank screen period, a fixation cross was displayed for 0.75 s before the stimuli were shown. During the practice trials, the inter-trial interval consisted only of the blank screen and a fixation cross, each lasting for 0.75 s.

**Figure 2:**
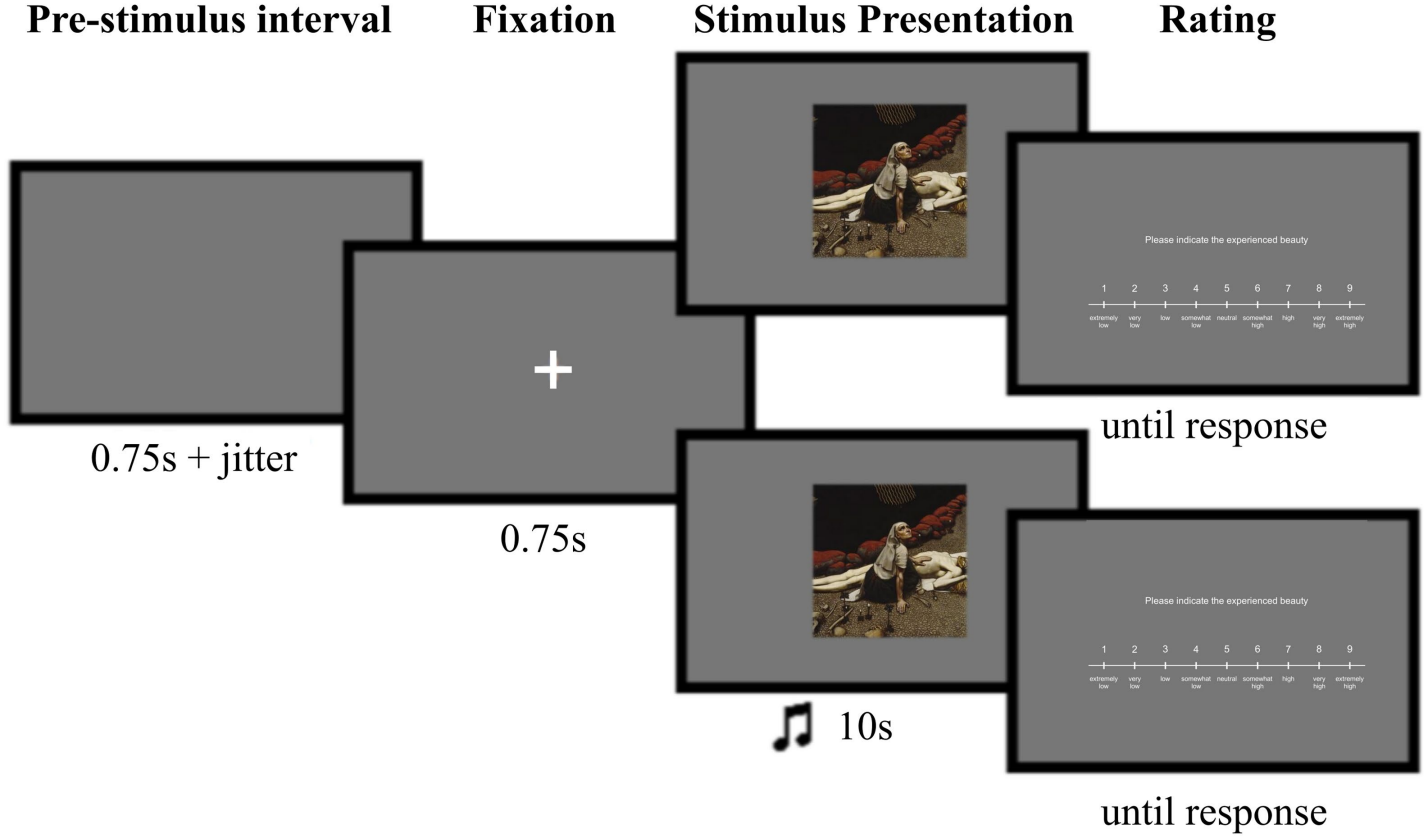
Layout of an experimental trial: either a unimodal condition (where a painting was shown alone—top), or a crossmodal condition (where a painting was presented with an emotionally congruent or incongruent music excerpt—bottom) (Wintermans, 2019). The first rectangle indicates the jittered Pre-stimulus interval, the second shows the Fixation period, followed by the Stimulus Presentation: the painting appeared in the upper part of the screen, with accompanying music added in the lower part during crossmodal trials. This was followed by a Rating response period in which participants rated the experienced beauty of the painting.

During each trial of the main experiment, after the fixation cross, a painting was displayed on the screen for 10 s, accompanied by either a music excerpt (crossmodal condition) or no sound (unimodal condition), depending on the trial type. After the presentation of the painting, participants were presented with a rating scale and used a button box with four buttons (HHSC-2×4-C, Current Designs) controlled by their right hand to rate the experienced beauty of the stimulus. The instruction presented on the screen was “Please indicate the experienced beauty,” and the rating scale was shown below (Figure 2). The Likert scale consisted of a 9-point scale ranging from “extremely low” to “extremely high.” A triangle above the scale indicated the position, and participants could move it left or right using the corresponding buttons. Once they were satisfied with the rating, they accepted it by pressing the green button on the button box. During the three breaks within each scanning session, participants had the freedom to decide when to continue.

Throughout the experiment, a mid-grey background was used. The screen was viewed with a visual angle of approximately 27.26° × 15.54°, and the paintings themselves were viewed with a visual angle of approximately 8.67° × 8.67°. The distance between the screen and the mirror was approximately 134 cm, while the distance between the mirror and the participants’ eyes was approximately 10 cm. The sound volume was adjusted to a comfortable level for each participant before the experiment began.

### 2.4 MRI data acquisition

The data acquisition process involved using a 3 T MAGNETOM PrismaFit MR scanner with a 32-channel head coil. The fMRI data were acquired using the multiband-4 (MB4) protocol, resulting in a 2.0 mm isotropic voxel size. The scanning parameters included a TR (Repetition Time) of 1.5 s, a TE (Echo Time) of 39 milliseconds, and a flip angle of 75°. Using the multiband-4 (MB4) acceleration factor, we captured images with 68 slices per volume, ensuring comprehensive brain coverage. The slices were acquired with no distance factor (0% slice gap), meaning there was no space between consecutive slices, allowing for a contiguous and precise representation of the brain structure. Each slice was 2.00 mm thick, matching our goal for isotropic voxel resolution.

The experiment consisted of two separate scanning sessions, with each session including four runs of the experiment. Each experimental run lasted between 17 to 20 min, depending on how quickly participants responded to the rating scale. For both of the two sessions, the total duration per session amounted to approximately 1.5 h. During the first scanning session, a structural scan was performed in the middle of the experiment after the first two runs, which lasted 5 min. The structural scan utilized the 3D Magnetization Prepared Rapid Acquisition Gradient Echo (MPRAGE) protocol, acquiring a T1-weighted image in the sagittal orientation. The structural scan had a voxel size of 1.0 mm isotropic, a TR of 2.30 s, a TI (Inversion Time) of 1.10 s, a TE of 3.03 milliseconds, and a flip angle of 8°. Parallel imaging (iPat = 2) was employed to accelerate the acquisition process.

### 2.5 Participants

Twenty eight participants who signed up for the experiment through the Radboud University student subject pool took part in the study and received compensation. To be able to detect an effect with a small-to-medium effect size for a more complex design, we initially recruited up to *N* = 28 participants. As the performed analyses focused on a reduced set of conditions, the resulting sample of *N* = 21 (*d* = 0.25, *α* = 0.05, and power = 0.8) was sufficient for the 2 × 2 design we report. The study consisted of two experimental sessions. However, the COVID-19 pandemic prevented Subjects 3, 8, 11, and 12 from participating in both sessions. Three subjects (16, 19, and 20) were excluded due to exceeding motion parameters, which led to poor fMRI data quality. Therefore, the final sample for analysis consisted of 21 participants who completed both sessions: 15 female and 6 male participants, with an average age of *M* = 25.71, *SD* = 6.16. All participants had normal or corrected-to-normal vision, normal hearing, and no color blindness. They did not report being claustrophobic, having epilepsy, having undergone brain surgery, having metal objects in or on their bodies (except for tattoos and dental wires), or being pregnant. Participants reported no history of psychiatric or neurological disorders. The written consent form was signed by participants before the study, which the ethics committee of Radboud University approved. Participants were compensated at an hourly rate of €10 for their time spent in the study.

### 2.6 Data analysis

#### 2.6.1 Behavioral analysis

For Research Question 1, we explored the impact of crossmodal presentation (auditory and visual) versus unimodal on participants’ overall beauty ratings of the paintings, manipulating the independent variables of Modality (Crossmodal vs. Unimodal) and Semantics (Original vs. Fourier Scrambled). Following exposure to the assigned stimuli, participants provided ratings for their overall liking/appreciation using a 9-point scale ranging from “extremely low” to “extremely high.” Subsequently, we conducted two separate 2×2 Repeated ANOVA analyses for each research question, assessing the main effects of the relevant independent variables. Research Question 2 examined the impact of emotional congruency/incongruency between the visual and auditory stimuli in the crossmodal conditions on subjective beauty ratings. Here, the independent variables were Congruency (Congruent vs. Incongruent) and Semantics (Original vs. Fourier Scrambled), with the conditions of Congruent Original, Congruent Fourier Scrambled, Incongruent Original, or Incongruent Fourier Scrambled. In the latter, we also examined the interaction effect of main factors. We used post-hoc tests to follow up on any significant differences.

#### 2.6.2 Data exclusion criteria

Subjects 16, 19, and 20 were excluded from the study due to excessive head motion beyond the −2 to +2 mm range, impacting data quality. Movements exceeding 1.5 or 2 mm thresholds can introduce artifacts, leading to exclusions based on previous research (Poldrack et al., 2011; Di and Biswal, 2023). After careful analysis, only run 3 for Participant 6 was omitted due to the presence of high motion parameters confined to a specific run. As a result, our dataset was ultimately refined to include data from 21 subjects for further analysis.

#### 2.6.3 Univariate fMRI analysis

In the study, univariate analyses of fMRI data were performed using a systematic approach. The preprocessing steps involved slice-time correction, realignment to correct for subject motion, coregistration, tissue-specific segmentation, normalizing the data to MNI space (Ashburner et al., 2014), and ensuring accurate alignment across participants and smoothing with a FWHM kernel of 8 mm. Next, a whole-brain General Linear Model (GLM) analysis was conducted for each participant individually. This analysis aimed to investigate the effects of the experimental manipulations by examining univariate contrasts.

During the GLM analysis, brain volumes were analyzed based on their association with specific trials. We performed first-level and second-level analyses using SPM software (SPM12, Ashburner et al., 2014). At the first level, individual-level GLM analyses were conducted, modeling the data for each participant. Our experimental design matrix incorporated 6 conditions, structured into regressors of original trials that encompassed both unimodal and crossmodal stimuli, each further categorized into congruent and incongruent conditions. Therefore, we allocated three regressors in the matrix for original trials and three additional regressors for trials involving Fourier Scrambled, alongside six regressors dedicated to capturing motion parameters and participant ratings for inclusion in the general linear model (GLM). This setup allowed us to isolate and contrast the effects of interest precisely. We included the duration of the stimulus beauty rating duration as a regressor to account for the variable delay caused by the time participants took to rate the stimuli, during which the rating scale, and not the painting, was presented on the screen. Therefore, the design matrix included the period when participants were actively observing and engaging with the experimental stimuli, both visual and auditory (10 s), as well as the duration of the period during which participants were providing subjective ratings for the stimuli.

The second-level analysis combined the statistical maps across participants, enabling the identification of common activation patterns and differences between conditions at the group level. In line with behavioral analysis, we performed fMRI contrasts for Research Question 1 as follows: Crossmodal (all Congruent Original, Congruent Fourier Scrambled, Incongruent Original, and Incongruent Fourier Scrambled trials) versus Unimodal (Original and Fourier Scrambled trials) and Crossmodal Original (Congruent + Incongruent trials) versus Unimodal Original. For Research Question 2, we performed several contrasts. Firstly, for a more general understanding of the emotional congruency effect, we performed the contrast between Congruent Original and Incongruent Original conditions as well as the contrast between Incongruent Original and Congruent Original conditions. Then, to assess the impact of low-level features, the contrast between Congruent Fourier Scrambled and Incongruent Fourier Scrambled conditions is performed. Lastly, for assessing the interaction effect between congruent and incongruent when the low-level features’ impact is controlled, the interaction contrast (Congruent Original versus Fourier Scrambled) - (Incongruent Original versus Fourier Scrambled) is performed.

## 3 Results

We first present the behavioral results (Section 3.1), followed by the fMRI results (Section 3.2).

### 3.1 Behavioral results

**Figure 3:**
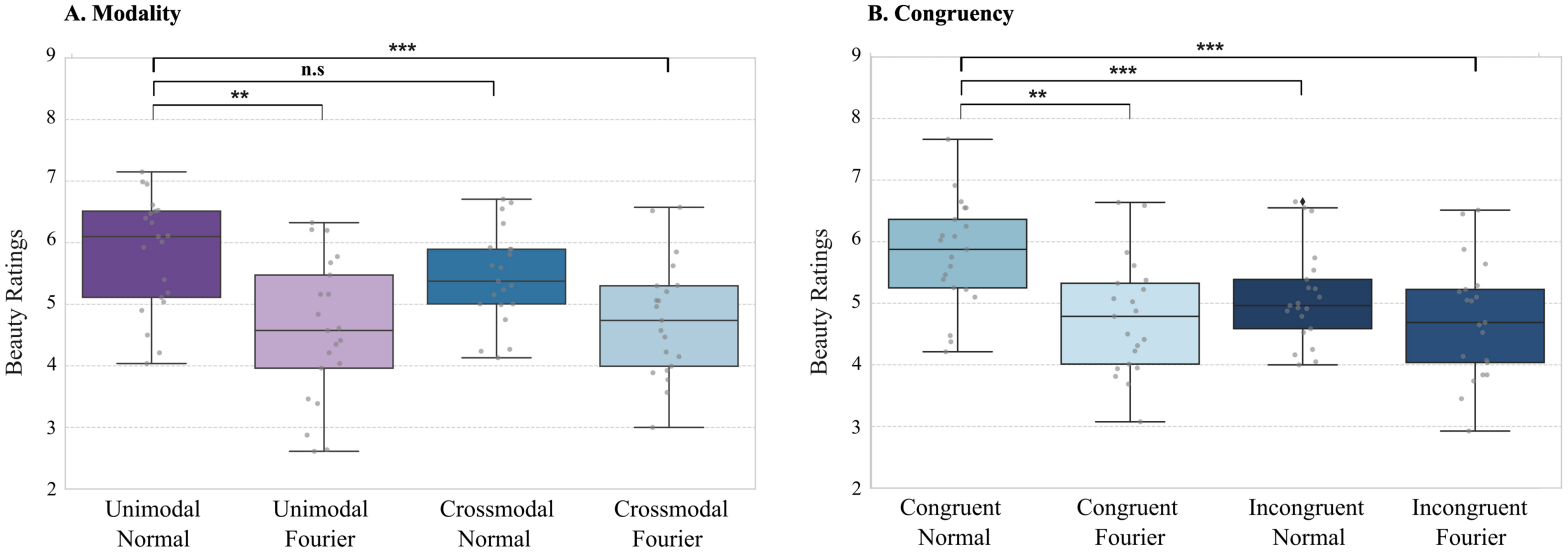
**(A)** Behavioral results of modality (Crossmodal vs. Unimodal) and semantics (Original vs. Fourier Scrambled). Boxplot of beauty ratings for modality conditions. Significant effects are indicated: the **semantics effect (**p = 0.0033) between Fourier Scrambled conditions and the ***modality × semantics interaction (**p = 0.0027) between Unimodal Original and Crossmodal Fourier Scrambled. **(B)** Behavioral results of emotional congruency/incongruency. Boxplot of beauty ratings for emotional congruence conditions. Significant effects are indicated: the **congruency effect (***p < 0.0001) between Congruent Original and Incongruent Original, the **congruency × semantics interaction (***p = 0.0001) between Congruent Original and Incongruent Fourier Scrambled, and the **semantics effect (***p = 0.0073) between Congruent Original and Congruent Fourier Scrambled. Error bars represent standard errors of the mean.

In Research Question 1, we explored the impact of unimodal versus crossmodal presentation on beauty ratings, as shown in Figure 3A. A 2×2 Repeated Measures ANOVA was conducted with Modality (Unimodal vs. Crossmodal) and Semantics (Original vs. Fourier Scrambled) as factors. In Research Question 2, Figure 3B presents the effect of emotional congruency/incongruency between auditory and visual stimuli on beauty ratings in the crossmodal conditions. A 2×2 Repeated Measures ANOVA was conducted with Congruency (Congruent vs. Incongruent) and Semantics (Original vs. Fourier Scrambled), also examining their interaction. Post-hoc tests were performed to follow up on significant effects.

In addition to the main analyses, an exploratory analysis was conducted to examine the influence of happy and sad valences on beauty ratings in congruent and incongruent crossmodal stimulus pairs. The results, presented in Supplementary Figure 4, showed that sad-sad congruent pairs were rated significantly higher in beauty than happy-happy pairs, while the emotional content of the auditory stimulus had a stronger influence in the incongruent conditions. For the incongruent condition, sad music-happy painting pairs had higher ratings than happy music-sad painting.

#### 3.1.1 The effect of modality (crossmodal versus unimodal)

The repeated measures ANOVA analysis of the beauty ratings indicated no significant main effect of Modality, *F*(1, 18) = 2.26, *p = 0.150*; a significant main effect of Semantics, F(1, 18) = 11.76, *p = 0.003*; and a significant interaction between Modality and Semantics, F(1, 18) = 11.08, *p* = *0.004*. (Figure 3A). Our findings showed no significant difference in beauty ratings between the Crossmodal Original and Unimodal Original trials (no main effect of Modality), likely due to the inclusion of both Emotionally Congruent and Incongruent trials in the Crossmodal condition. As can be seen in Figure 3B, the effect of emotional (in)congruency in crossmodal trials may be due to the inclusion of both Congruent and Incongruent conditions. Furthermore, it can be seen in Figure 3A that beauty ratings for Fourier Scrambled stimuli were generally lower than for original paintings (main effect of Semantics), indicating that the presence of semantic information generally enhanced the beauty ratings and showed no distinction between the two modalities.

Through *post hoc* analyses using Tukey’s HSD, we investigated the impact of Modality (Crossmodal vs. Unimodal) and Semantics (Original vs. Fourier Scrambled) on the significance of the interaction effect. The analysis did not reveal significant differences between Crossmodal and Unimodal conditions within either Original-only or Fourier Scrambled-only trials. However, when delving into the Original vs. Fourier Scrambled comparisons (effect of semantics) across different levels of Modality, notable differences emerged. Specifically, the Original_Unimodal condition demonstrated significantly higher beauty ratings compared to Fourier Scrambled_Crossmodal (mean difference = 1.167, *p = 0.002*), and Original_Crossmodal beauty ratings were significantly higher than those in Fourier Scrambled_Unimodal (mean difference = 0.9289*, p = 0.0203*). Additionally, a significant difference favored Original_Unimodal over Fourier Scrambled_Unimodal (mean difference = 1.3204*, p < 0.001*). On the contrary, Fourier Scrambled_Crossmodal vs. Original_Crossmodal did not reveal any significant outcome. The contrast between Fourier Scrambled_Crossmodal and Fourier Scrambled_Unimodal, as well as the contrast between Fourier Scrambled_Crossmodal and Original_Crossmodal, revealed no significant difference. These findings suggest that both semantic clarity and emotional congruency influence interaction effects, demonstrating that paintings with intact information generally receive higher beauty ratings. Specifically, semantic clarity enhances beauty ratings, as seen in the higher ratings for Original compared to Fourier Scrambled stimuli, particularly in the unimodal condition. However, in the crossmodal condition, emotional congruency further modulates these effects, with congruent pairings enhancing beauty ratings and incongruent pairings reducing them. This suggests that both semantic information and perceived congruency contribute to aesthetic experience in distinct yet interacting ways.

#### 3.1.2 The effect of emotional (in)congruency

The behavioral results of the beauty ratings indicated a significant main effect of Congruency, *F*(1, 18) = 27.34, *p < 0.001*; a significant main effect for Semantics, F(1, 18) = 10.07, *p = 0.005*, and a significant interaction between Congruency and Semantics, F(1, 18) = 20.95, *p < 0.001* (Figure 3B). Consistent with our hypothesis, Congruent Original trials yielded higher beauty ratings than their incongruent counterparts. Meanwhile, beauty ratings for Fourier Scrambled trials demonstrated little difference, remaining relatively consistent. This could be caused by the effect of semantics, where Original trials are figurative paintings containing semantic cues that elicit emotions. At the same time, Fourier Scrambled has only color information with low-level features, leading to higher beauty ratings in Original trials compared to Fourier Scrambled, which is consistent with Crossmodal versus Unimodal behavioral beauty rating comparisons.

### 3.2 fMRI results

**Figure 4:**
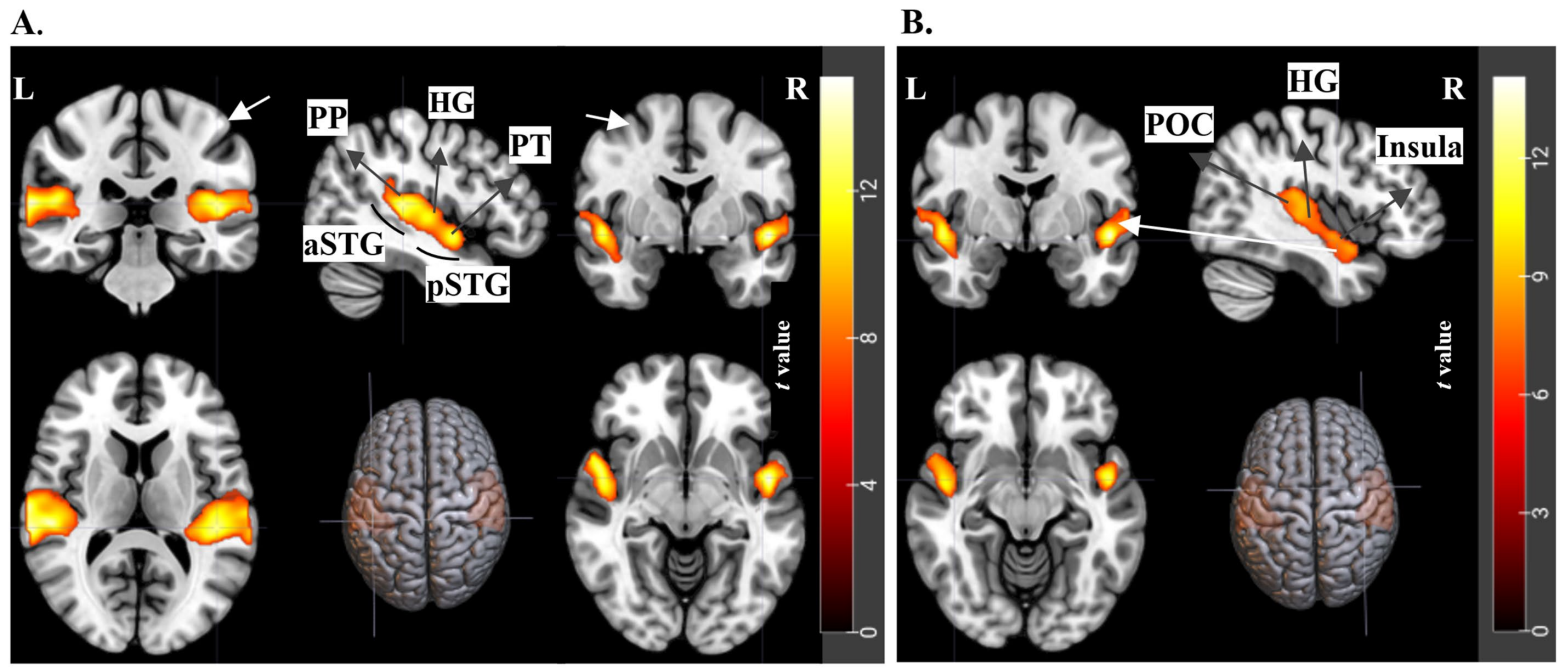
fMRI results for the contrast of Crossmodal Original > Unimodal Original trials. t-statistics for 2nd level analysis, *N* = 21 subjects, with a threshold of FWE *< 0.05* and cluster size>20. **(A)** Shows auditory areas such as the Planum Polare (PP), Heschl’s Gyrus (HG), Planum Temporale (PT), and both the anterior (aSTG) and posterior (pSTG) portions of the Superior Temporal Gyrus. **(B)** Highlights Parietal Operculum Cortex (POC), Heschl’s Gyrus (HG), and Insula in the sagittal plane, with detailed views of the Insula.

**Figure 5:**
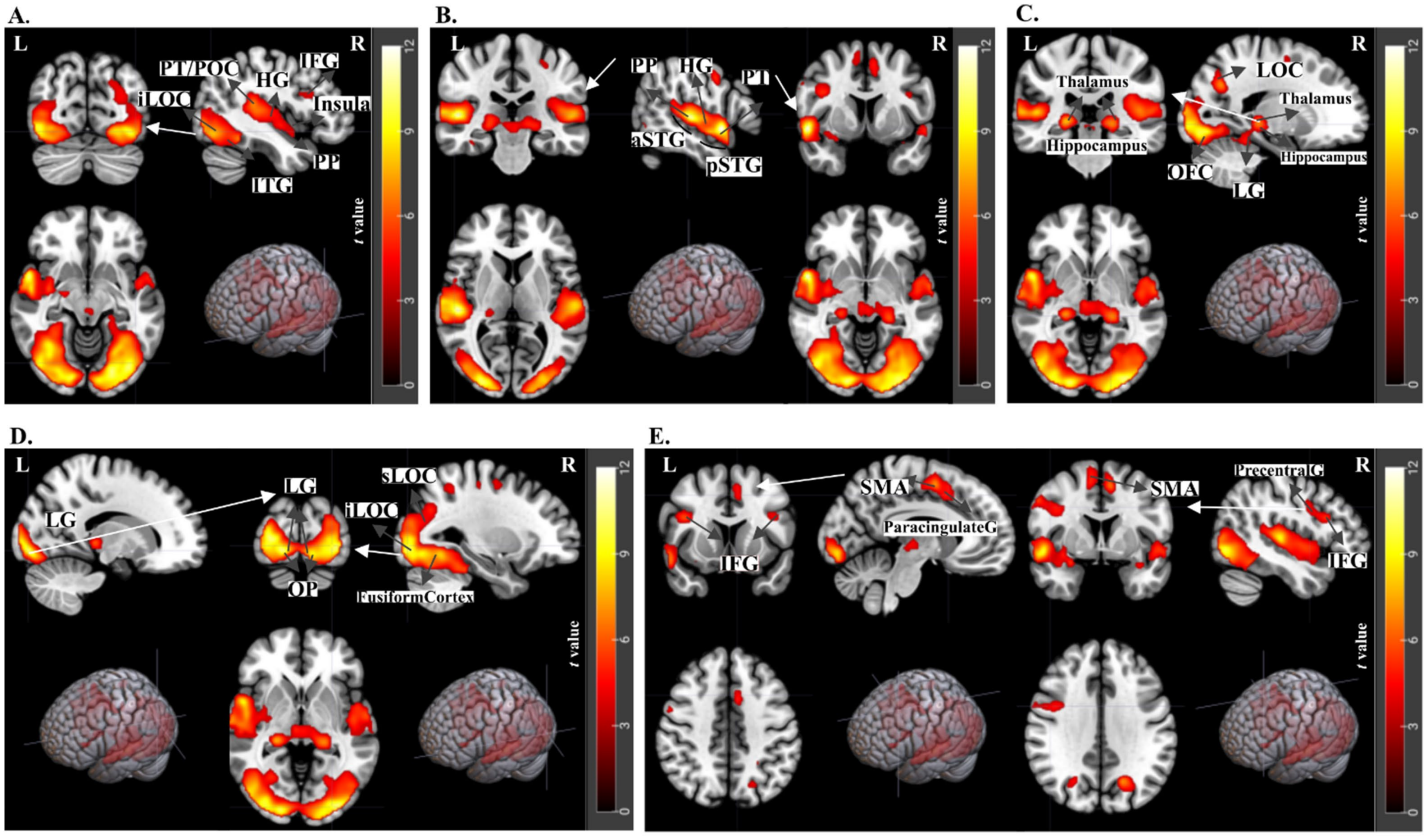
fMRI results for emotional congruency. t-statistics for 2nd level analysis, *N* = 21 subjects, with a threshold of p-uncorrected < 0.001 and cluster size>20. Congruent Original versus The Incongruent Original contrast revealed significant activations at **(A)** Occipital areas, including inferior Lateral Occipital Cortex (iLOC), Inferior Temporal Gyrus (ITG), and auditory areas Heschl’s Gyrus (HG), Planum Polare (PP), Planum Temporale (PT), Parietal Operculum Cortex (POC), as well as frontal lobe activations IFG, Inferior Frontal Gyrus (IFG). Additional activations at Insula. **(B)** Auditory areas Heschl’s Gyrus (HG), Planum Polare (PP), Planum Temporale (PT) **(C)** Thalamus, Hypothalamus activations as well as Occipital Fusiform Gyrus, Lingual Gyrus, and Lateral Occipital Cortex **(D)** The ventral stream of the occipital cortex for visual processing areas such as the Lingual Gyrus, Occipital Pole, inferior and superior portions of the Lateral Occipital Cortex, and Fusiform Cortex at both occipital and temporal lobes **(E)** Inferior Frontal Gyrus, Juxtapositional Lobule Cortex (formerly Supplementary Motor Cortex - SMA), Precentral Gyrus, and Paracingulate Gyrus.

**Figure 6:**
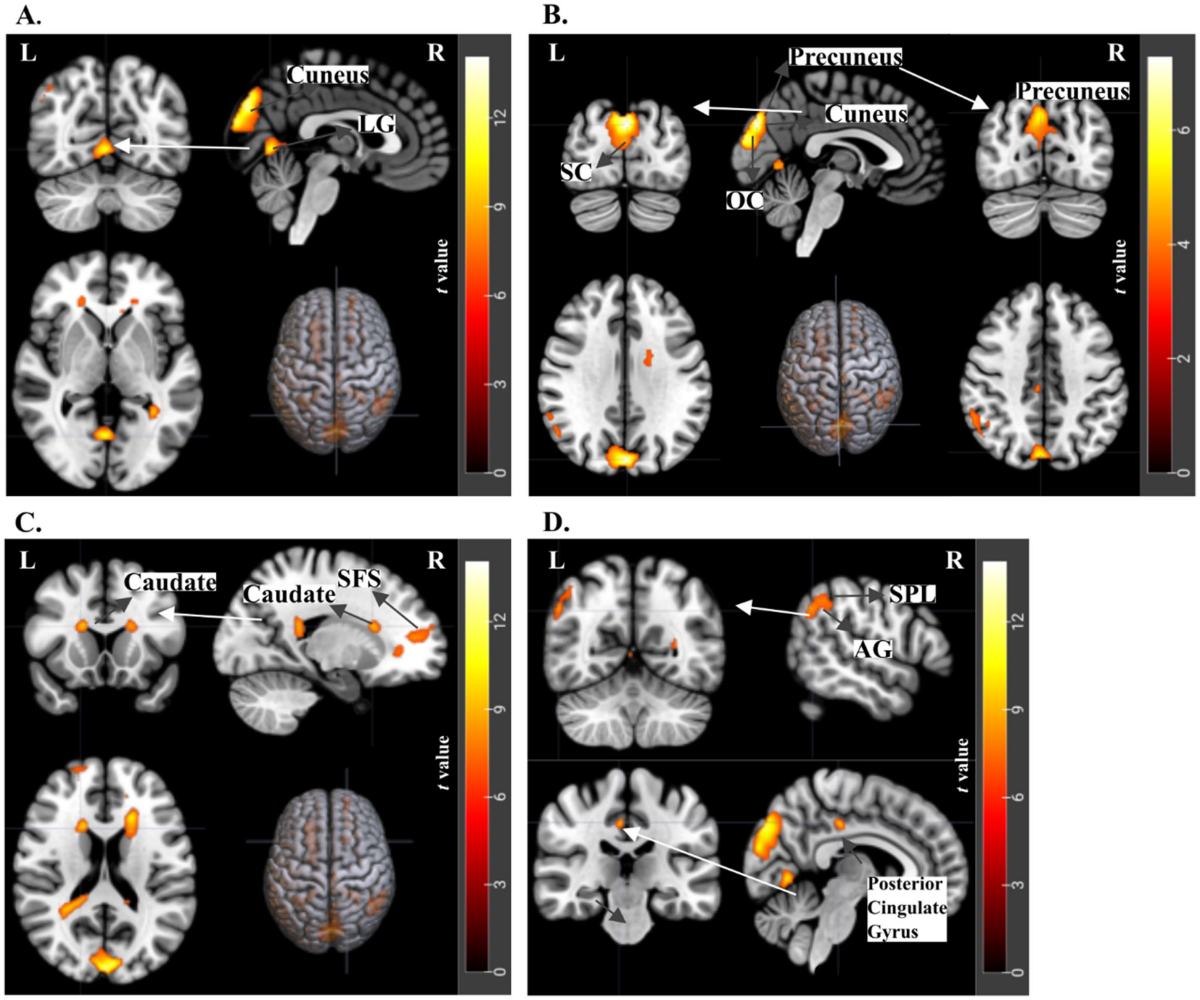
fMRI results for emotional incongruency. t-statistics for 2nd level analysis, *N* = 21 subjects, with a threshold of p-uncorrected < 0.001 and cluster size>20. The Incongruent Original versus Congruent Original contrast revealed significant activations at **(A)** Cuneus and Lingual Gyrus, **(B)** Cuneus, Precuneus, Supracalcarine Cortex (SC), and Occipital Cortex (OC), **(C)** Caudate and Superior Frontal Sulcus **(D)** Superior Parietal Lobe and Angular Gyrus (top) and Posterior Cingulate Gyrus (bottom).

**Figure 7:**
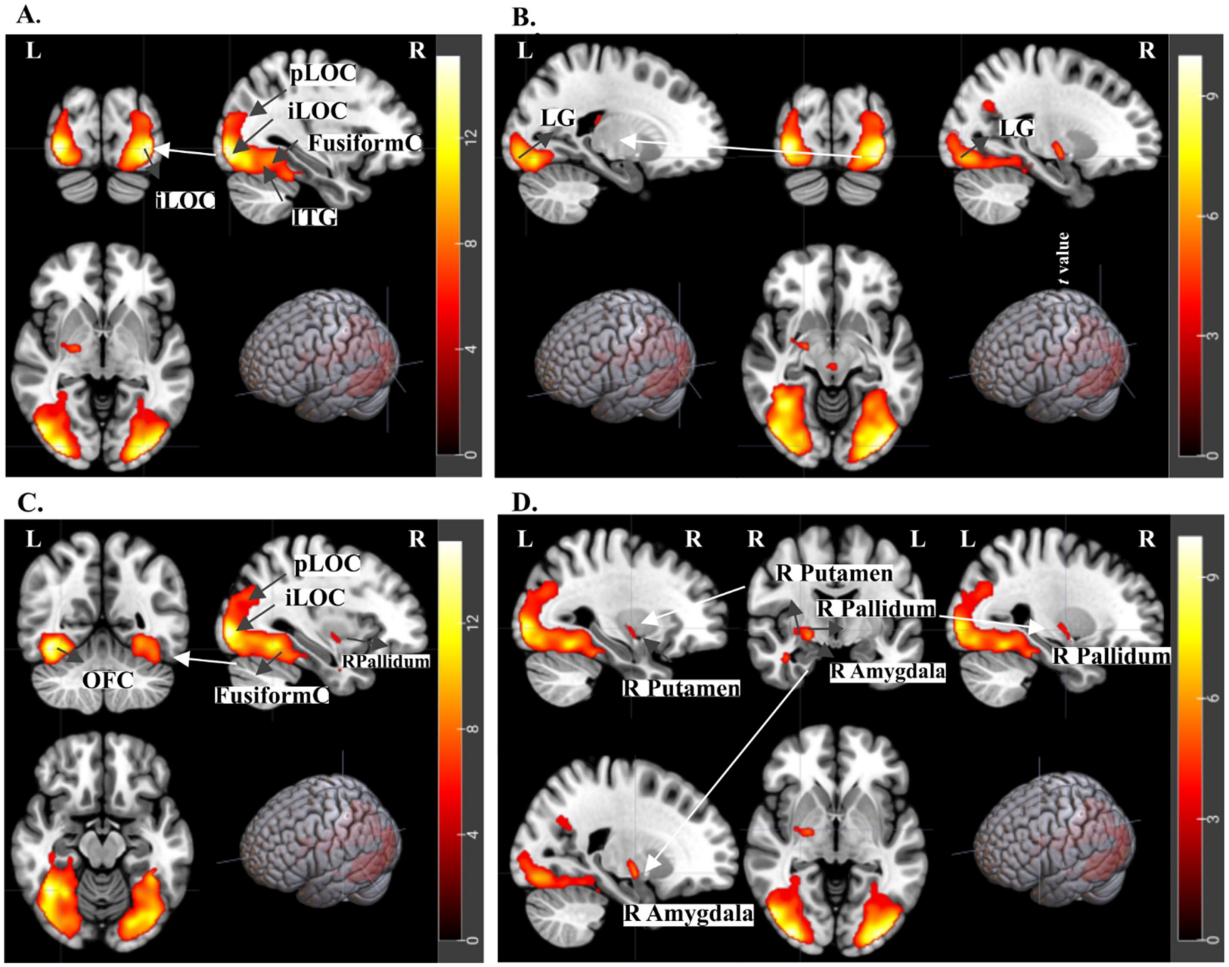
fMRI results for Interaction Effect: (Congruency Original-Fourier Scrambled) - (Incongruency Original-Fourier Scrambled) t-statistics for 2nd level analysis, *N* = 21 subjects, with a threshold of p-uncorrected < 0.001 and cluster size>20. **(A)** The ventral stream activations of the occipital cortex for visual processing areas such as anterior and posterior portions of the Lateral Occipital Cortex and Fusiform Cortex at both occipital and temporal lobes, as well as Inferior Temporal Gyrus **(B)** Revealing activations at Lingual Gyrus (LG), zoomed in on the Axial and Coronal planes. **(C)** The ventral stream of the occipital cortex for visual processing areas, such as the anterior and posterior portions of the Lateral Occipital Cortex and Fusiform Cortex at both occipital and temporal lobes, as well as the R Pallidum. **(D)** Right Putamen, Right Pallidum, and Amygdala as well as the inferior longitudinal fasciculus (ILF).

**Figure 8:**
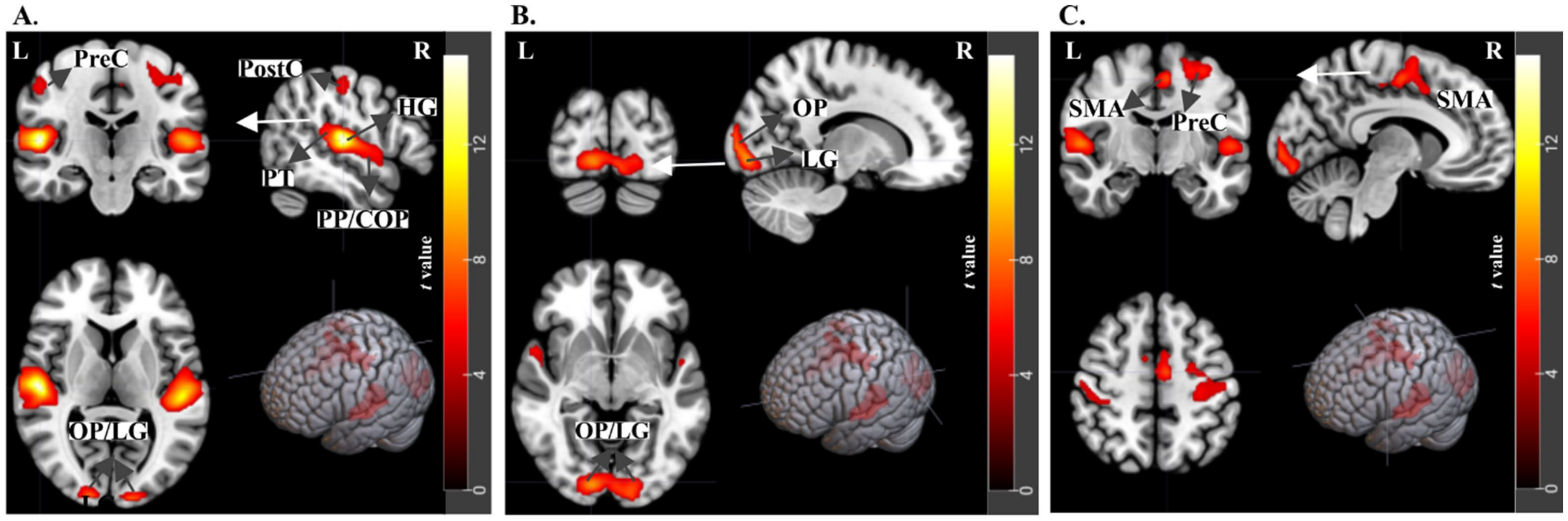
fMRI results for Congruent Fourier Scrambled > Incongruent Fourier Scrambled contrast. t-statistics for 2nd level analysis, *N* = 21 subjects, with a threshold of p-uncorrected < 0.001 and cluster size>20. **(A)** Auditory activations at the Heschl’s Gyrus (HG), Planum Temporale (PT), Planum Polare (PP), Central Operculum Cortex (COP), as well as Precentral and Postcentral Gyrus (PreCG and PostCG). Further activations at Occipital Pole (OP) and Lingual Gyrus (LG). **(B)** Revealing activations at Occipital Pole (OP) and Lingual Gyrus (LG). **(C)** Demonstrating activations at the Juxtapositional Lobule Cortex (formerly Supplementary Motor Cortex - SMA) and Precentral Gyrus (PreCG).

In visualizing the fMRI results, each figure (Figures 4–8) showcases sagittal, axial, and coronal images along with rendered views. These images are annotated to highlight significant activations, using white arrows and labeled abbreviations to denote key brain areas of interest.

The confirmatory analysis of the overall effects of modality and semantics included all trials: Modality - Crossmodal versus Unimodal, and Semantics - Original versus Fourier Scrambled. The fMRI results, related coordinate tables are provided in Supplementary Figures 1, 2, and Supplementary Table 1. In these broader comparisons (since they include all trials in two conditions – Crossmodal versus Unimodal and Original versus Fourier Scrambled), fMRI results for Crossmodal versus Unimodal revealed significant activation in auditory processing areas, including the STG, Planum Polare, Heschl’s gyrus, and Planum Temporale. Additionally, when analyzing Original versus Fourier Scrambled paintings, we found activations in the LOC, Fusiform Gyrus, and Thalamus, highlighting the importance of semantic content for processing objects and faces. These findings support the distinction between trials with and without semantic information. Together, these analyses validate our experimental design by confirming the main effects of modality and semantics.

#### 3.2.1 The effect of modality (crossmodal versus unimodal)

To specifically examine modality effects, this section focuses exclusively on original paintings, excluding the Fourier Scrambled trials, as they seemed not to contribute substantially to the results. This decision was based on the findings outlined in the Supplementary section, where we compared crossmodal and unimodal conditions using both Original and Fourier Scrambled data. As the results in Supplementary Figure 1, Supplementary Table 1 show that the Fourier Scrambled trials did not contribute significantly to the observed effects, we have chosen to present only the Original comparison in this section. For the contrast of Crossmodal Original versus Unimodal Original, the fMRI results showed significant activation in auditory regions (Figure 4, Table 2). Specifically, the Planum Polare (PP), Heschl’s Gyrus (HG), Planum Temporale (PT), and both anterior and posterior segments of the Superior Temporal Gyrus (aSTG and pSTG) were more active for crossmodal versus unimodal trials. Additionally, the Parietal Operculum Cortex (POC) and the Insula were highlighted, showing their involvement. Comparing Supplementary Figure 1 with Figure 4 below reveals no observable difference in activated brain regions between the broader Crossmodal to Unimodal contrast (including both Original and Fourier Scrambled trials) and the more focused Crossmodal Original versus Unimodal Original comparison. These results indicate that the activation is mainly driven by both the inclusion of music (modality effect) and semantic content, and not changed by adding the Fourier Scrambled trials.

#### 3.2.2 The effect of emotional (in)congruency

In this section, first, we compare congruent versus incongruent conditions to examine emotional congruency in Original trials only, similar to the approach in previous studies, focusing only on original images. Secondly, we present the reverse comparison - emotional incongruency. To extend beyond the emotional (in)congruency contrasts, the interaction effect of congruency versus semantics provides a clearer distinction between congruency and incongruency by controlling for low-level features, ensuring that emotional congruency effects are driven by semantic information (Congruency Original-Fourier Scrambled - Incongruency Original-Fourier Scrambled). For more general (confirmatory) main contrasts regarding emotional (in)congruency, we have added the fMRI results and corresponding tables in the Supplementary: Original versus Fourier Scrambled (Supplementary Figure 2, Supplementary Table 1 main effect of semantics) and the main contrast of Congruency (Original + Fourier Scrambled) versus Incongruency (Original + Fourier Scrambled) (Supplementary Figure 3, Supplementary Table 3).

##### 3.2.2.1 The contrast for emotional congruency (congruent versus incongruent trials)

For the effect of emotional congruency, we used different thresholds for the fMRI results. It is the case that when comparing across modalities, the Crossmodal condition included both Congruent and Incongruent trials (each of 80 trials), resulting in a higher number of trials (in total of 160 trials). Consequently, for general contrasts like the effect of Modality (including Crossmodal versus Unimodal and Original versus Fourier Scrambled comparisons in the Supplementary materials), we applied a threshold of family-wise error (FWE) correction for multivoxel comparisons. However, for specific contrasts such as Emotional Congruency and Incongruency, which contained fewer trials, we used p-uncorrected thresholds with clusters exceeding 50 voxels. We report the corresponding uncorrected *p*-values for the activated brain areas in Figures 5–8 and Tables 3–5. In these reports presented in Tables 3–5, significant results surviving FDR correction (*p* < 0.05) are marked with an asterisk (*).

**Table 3:** Cluster characteristics and coordinates for the contrast of Crossmodal Original versus Unimodal Original trials.

Region	Cluster size	Peak coordinates (MNI)			Z score
		x (mm)	y (mm)	z (mm)	
Crossmodal Original > Unimodal Original (*FWE<0.05*)					
Planum temporale/ Parietal Operculum Cortex/ Posterior STG	2,617	52	−28	12	6.69
Planum Polare/ Central Opercular Cortex		64	−30	12	6.66
Planum temporale/ Posterior STG		66	−18	10	6.62
Planum polare/ Anterior STG	2,373	−50	−2	−8	6.79
Planum temporale/ Heschl’s Gyrus		−50	−28	6	6.56
Planum temporale/ Posterior STG		−62	−22	8	6.21

**Table 4:** Cluster characteristics and coordinates for emotional congruency.

Region	Cluster size	Peak coordinates (MNI)			Z score
		x (mm)	y (mm)	z (mm)	
Congruency Original > Incongruency Original (*p < 0.001*)					
Occipital pole/ Lingual gyrus*	12,110	16	−90	−6	6,02
Lateral occipital cortex-inferior*		−34	−88	−4	6,00
R Thalamus/ R Hippocampus*		−38	−76	−10	5,99
Temporal Pole/Planum Polare*	3,460	54	6	−8	6,38
Planum temporale, Superior temporal gyrus – posterior*		52	−24	4	6,31
Superior temporal gyrus – anterior*		52	−4	−10	5,56
Planum temporale/ Heschl’s gyrus*	2,484	−58	−20	6	5,43
Parietal Operculum Cortex*		−46	−34	14	4,86
Superior temporal gyrus – anterior*		−60	−6	−4	4,63
Precentral gyrus*	523	58	2	40	4,47
Inferior frontal gyrus*		40	12	24	4,42
Precentral gyrus*		40	2	32	3,86
Juxtapositional lobule cortex/ precentral gyrus*	412	−8	0	54	4,95
		−8	10	44	3,87
Precentral gyrus*	190	−36	−10	52	3,53
		−22	−6	56	3,48
Juxtapositional lobule cortex/ Superior frontal gyrus	181	10	2	56	4,04
Superior frontal gyrus/ Middle frontal gyrus	119	−40	14	24	3,52
Superior parietal lobule	108	−26	−50	50	3,74
Superior parietal lobule/ Angular gyrus	80	30	−46	50	3,63
Lateral occipital cortex - posterior	73	24	−66	32	3,80

Results marked with * indicate significance after FDR correction (*p* < 0.05).

**Table 5:** Cluster characteristics and coordinates for emotional incongruency.

Region	Cluster size	Peak coordinates (MNI)			Z score
		x (mm)	y (mm)	z (mm)	
Incongruency Original > Congruency Original (*p < 0.001*)					
Cuneus cortex/precuneus*	1,105	−2	−82	32	4.98
		0	−92	20	4.66
L inferior occipito-frontal fasciculus / Corpus callosum	547	−22	28	14	4.42
		−18	10	22	3.93
Angular gyrus / Superior lateral occipital cortex*	323	54	−60	38	3.89
		62	−44	36	3.75
Caudate*	302	22	−42	24	3.99
		28	−46	20	3.93
Cuneus*	175	−34	−50	2	4.15
		−18	−42	14	4.14
Frontal pole*	174	18	48	12	3.60
		20	36	2	3.41
		22	60	20	3.22
Lingual gyrus*	161	2	−66	0	4.38
		2	−54	4	3.33
R caudate*	66	16	18	20	4.12
Cingulate gyrus	33	6	−26	40	3.87

Results marked with * indicate significance after FDR correction (*p* < 0.05).

In our analysis of emotional congruency, the fMRI results for emotional congruency contrasting Congruent Original with Incongruent Original trials revealed that there are significant activations for Congruent Original compared to Incongruent Original across various brain regions, indicating a complex neural response to audiovisual emotional processing (Figure 5, Table 4). Notably, significant activation was observed in the occipital areas, including the inferior Lateral Occipital Cortex (iLOC) and the Inferior Temporal Gyrus (ITG) (Figure 5A), indicating the neural engagement in visual processing (Cichy et al., 2011 and Baldauf and Desimone, 2014). Auditory areas also showed pronounced activation, with Heschl’s Gyrus (HG), Planum Polare (PP), Planum Temporale (PT), and the Parietal Operculum Cortex (POC) being implicated (Figures 5B,D), alongside activation in the Inferior Frontal Gyrus (IFG), underscoring the involvement of auditory processing and integration in emotional congruency (Beauchamp et al., 2004; Hein and Knight, 2008; Obleser et al., 2006). In the Crossmodal versus Unimodal comparison, we observed similar auditory activations in the STG; however, emotional congruency resulted in broader activations, extending to visual and frontal areas.

Further activations were observed in the Insula, a region associated with emotional processing (Sepulcre et al., 2012; Eickhoff et al., 2010), and the ventral stream of the occipital cortex, associated with visual processing, including the Lingual Gyrus, Occipital Pole, and the anterior and posterior portions of the Lateral Occipital and Fusiform Cortex (Kravitz et al., 2013; Pehrs et al., 2015). This pattern of activation extends to the Thalamus and Hypothalamus (Figure 5C). Additionally, the Occipital Fusiform Gyrus, along with the Lingual Gyrus and Lateral Occipital Cortex, were significantly activated, indicating these regions are involved in visual and emotional processing.

Moreover, the Inferior Frontal Gyrus, Juxtapositional Lobule Cortex (formerly known as the Supplementary Motor Cortex - SMA), Precentral Gyrus, and Paracingulate Gyrus showed significant activation (Figure 5E). Together, these results in processing emotionally congruent stimuli show activations spanning from primary sensory areas to higher-order cognitive and emotional processing centers.

##### 3.2.2.2 The contrast for emotional incongruency (incongruent versus congruent trials)

Neural activations contrasting Incongruent Original with Congruent Original conditions were observed in the Cuneus and Precuneus, alongside the Supracalcarine Cortex (Figures 6A,B). These areas are known to be involved in visual processing and attentional mechanisms (Kravitz et al., 2013; Seijdel et al., 2024). Additional activation was found in the Lingual Gyrus (Figure 6A), which is a higher-level visual processing region. Additionally, the Caudate and Superior Frontal Sulcus were significantly activated (Figure 6C), areas which are involved in cognitive control and emotional regulation. Moreover, significant activations were noted in the Superior Parietal Lobe and Angular Gyrus (Figure 6D), which are associated with spatial attention and the processing of emotional incongruency. The Posterior Cingulate Gyrus, known for its role in internally directed thought and emotional valuation, also showed significant activation. These results demonstrate a network of brain regions that are activated in processing emotionally incongruent stimuli (Table 5).

##### 3.2.2.3 Interaction effect of semantic congruency on cortical processing

This section focuses on the key aspect of this study: the interaction effect between emotional congruency and incongruency in the context of semantic associations between music and paintings (Figure 7, Table 6). Emotional congruency results highlighted the differences between congruent and incongruent conditions in original paintings rich in semantic content, designed to evoke more robust emotional responses when controlled for low-level visual features like color effects. The fMRI findings for the interaction effect revealed significant activations within the ventral stream, encompassing the Lateral Occipital Cortex (LOC), Fusiform Cortex, and Inferior Temporal Gyrus (shown in Figures 7A,C). Additionally, activation in the high-level visual processing area, the Lingual Gyrus, was observed (Figure 7B). Emotion-processing regions, including the Putamen, Pallidum, and Amygdala in the right hemisphere, were notably activated in this contrast (Figure 7D). This suggests enhanced emotional and visual processing during emotional congruency, aligning with our hypothesis.

**Table 6:** Cluster characteristics and coordinates for interaction effect: (Congruency Original-Fourier Scrambled)—(Incongruency Original-Fourier Scrambled).

Region	Cluster size	Peak coordinates (MNI)			Z score
		x (mm)	y (mm)	z (mm)	
Interaction effect: (Congruency Original-Fourier Scrambled)—(Incongruency Original-Fourier Scrambled) (*p < 0.001*).					
Temporal occipital fusiform cortex / Inferior temporal cortex*	4,855	42	−54	−14	5,87
		34	−88	−2	5,86
		50	−72	−4	5,80
Inferior lateral occipital cortex*	4,145	−38	−80	−10	5,84
		−34	−90	−2	5,77
		−42	−84	0	5,69
R Pallidum/ amygdala/ putamen*	128	22	−8	−8	4,54
		32	−4	−8	3,21

Results marked with * indicate significance after FDR correction (*p* < 0.05).

#### 3.2.3 Effect of low-level features: Fourier scrambled

The Fourier Scrambled versions of the paintings served to control for the influence of low-level features, which are color and brightness. In this analysis, we aim to confirm that the activations observed with the Fourier Scrambled images genuinely reflect low-level features and that we are not overlooking any significant unexpected activations. Consequently, Section 3.3 will primarily focus on comparing congruency and incongruency in original paintings, highlighting the key differences when semantic information is kept differently from Fourier Scrambled trials. For the Congruent Fourier Scrambled versus Incongruent Fourier Scrambled comparison, fMRI data revealed activations in sensory-related regions for both auditory and visual stimuli, including Heschl’s Gyrus (HG), Planum Temporale (PT), Planum Polare, Occipital Pole, and Lingual Gyrus. Furthermore, activations in the Juxtapositional Lobule Cortex (previously known as the Supplementary Motor Cortex - SMA) and the Precentral Gyrus (PreCG) were noted (Figures 8A–C), which may be related to the processing of low-level visual features (Binder et al., 2017). The table containing coordinate information and relevant details can be found in the Supplementary Table 2.

## 4 Discussion

This fMRI study examined how emotional congruency and incongruency between auditory (music) and visual (paintings) inputs affect experienced beauty, exploring the underlying neural mechanisms. Our key findings indicate that emotional congruency enhanced beauty ratings, while incongruency did not. When comparing congruent and incongruent crossmodal conditions, fMRI results revealed stronger brain activations in higher-order visual areas and emotion processing areas. Furthermore, the fMRI results indicating activations in cuneus, precuneus, and caudate were particularly interesting for emotional incongruency. Most importantly, the fMRI findings on the interaction effect for emotional congruency, with color and brightness controlled, revealed activations in the ventral stream, suggesting potentially in-depth processing of the paintings. These results provide valuable insights into the neural mechanisms underlying emotional (in)congruency, consistent with the beauty ratings.

### 4.1 Beauty ratings

When assessing the crossmodal trials for effects of emotional congruency (congruent vs. incongruent) and semantic content (Original vs. Fourier Scrambled), paintings in the emotionally congruent condition received higher beauty ratings than those in the incongruent condition. Previous behavioral studies have investigated whether congruency intensifies emotional ratings but not beauty (Jeong et al., 2011; Müller et al., 2011). Other studies have used congruency of features like complexity and regularity rather than emotion. For example, Rančić and Marković (2019) combined abstract paintings with jazz music based on these perceptual dimensions. They found that while congruence between music and paintings enhanced the perceived correspondence in terms of regularity and complexity, it did not significantly influence aesthetic preference. A recent study by Fink et al. (2024) explored how congruence between music and paintings influences aesthetic experience, using curated audiovisual pairs based on emotional tone and artistic style. While curated pairs were rated as more corresponding, no differences emerged in viewing time or aesthetic appreciation. Although Fink’s study and ours both use music-painting pairs, the key difference lies in how congruency is defined: Fink et al. (2024) focuses on curated versus random pairings, whereas our study specifically investigates emotional congruency, distinguishing between happy and sad stimuli. Distinct from these studies that focused on non-emotional features or broader congruency definitions, our results suggest that when modalities are matched emotionally, congruency enhances perceived correspondence and impacts aesthetic appreciation (beauty). This may be due to using emotionally congruent pairs and artistic stimuli, which elicited higher ratings for experienced beauty, and might be associated with emotional mediation correspondence (Spence, 2020). It is known that crossmodal correspondences enhance multisensory integration by aligning sensory inputs in a meaningful way (Parise and Spence, 2013), e.g., by improving response speed and accuracy in temporal and spatial judgments (Parise and Spence, 2009). In the context of emotional experiences, such crossmodal emotional correspondences may mediate aesthetic appreciation, with emotionally congruent pairings enhancing perceived beauty, while incongruent pairings disrupt this effect, leading to lower ratings.

### 4.2 Emotional congruency versus incongruency

Our fMRI findings on emotional congruency versus incongruency in the crossmodal condition highlighted significant activations across distinct brain regions, categorizing them into sensory processing, emotional processing, and cognitive processing centers. Sensory processing areas, such as the occipital regions (including the inferior LOC, Lingual Gyrus, and Inferior Temporal Gyrus) and auditory regions (Heschl’s Gyrus, Planum Polare, and Planum Temporale) were prominently activated, indicating robust engagement in visual and auditory processing, respectively (Kravitz et al., 2013; Cichy et al., 2011; Belardinelli et al., 2004; Baldauf and Desimone, 2014; Robins et al., 2009). Similar to the studies (Petrini et al., 2011; Sepulcre et al., 2012; Eickhoff et al., 2010), we observed activations in the Insula, Thalamus, and Hypothalamus regions that might be associated with emotional processing. The activations that we observed in the frontal lobe regions, such as the Inferior Frontal Gyrus, Precentral Gyrus, and Paracingulate Gyrus, could be indicative of cognitive processes related to emotional congruency that were also found in these studies (Gao et al., 2020; Kreifelts et al., 2009). These findings collectively suggest that emotionally congruent stimuli elicit stronger widespread activations across several brain regions involved in sensory integration, emotional processing, and cognitive processing compared to incongruent stimuli.

In our data, when comparing emotional congruency versus incongruency using original paintings with semantic content while controlling for color and brightness through Fourier Scrambled images, we observed strong activation in the ventral stream. Notably, our results indicate that congruent audiovisual emotional stimuli may enhance higher visual processing compared to incongruency in these object recognition areas, including the LOC and face-selective regions like the fusiform gyrus, both of which are key components of the ventral stream. Research has shown that top-down attention modulates how audiovisual stimuli are integrated (Talsma et al., 2010; Seeley, 2012; Gao et al., 2023). Previous studies (Seijdel et al., 2024; Gerdes et al., 2021) suggest that the perception and processing of congruent audiovisual stimuli may be enhanced when attention is directed toward emotionally relevant aspects. The allocation of attention through congruency might enhance the detailed processing of elements within the paintings, such as objects, scenes, and faces. Thus, the activation in the ventral stream may reflect the detailed processing facilitated by attentional resources, suggesting a potential role of attention in our findings. Therefore, our findings lead us to speculate that the emotionally congruent music played with the paintings may have enhanced attention to the paintings, resulting in the observed activation patterns, and suggesting that emotional auditory cues can guide visual attention toward emotionally relevant stimuli.

### 4.3 Emotional incongruency versus congruency

While emotional congruency is relatively well understood in the literature (e.g., Jansma et al., 2014; Klasen et al., 2011; Müller et al., 2011; Dolan et al., 2001; Petrini et al., 2011), emotional incongruency remains relatively understudied and warrants further attention. It is a complex process, involving conflicting visual and auditory stimuli. Some neural findings regarding incongruence are either contradictory (Müller et al., 2011; Klasen et al., 2011) or inconclusive (Dolan et al., 2001). For instance, Dolan et al. (2001) contrasted emotionally congruent with emotionally incongruent conditions in an audiovisual paradigm. They observed greater activation of the left amygdala and right fusiform gyrus (FFG) in congruent conditions compared to incongruent ones, but did not report a significant effect in the reverse contrast. These inconsistencies show the challenges in capturing the effects of emotional incongruency. In addition to these studies, our research identified important neural areas involved in emotional incongruency, such as cuneus, precuneus, and caudate, contributing to a deeper understanding of emotional congruency in the context of existing literature.

For Incongruent Original versus Congruent Original trials, we report stronger activation in occipital areas, including the cuneus and supracalcarine cortex, as well as the precuneus in the parietal region. The cuneus and supracalcarine cortex are primarily associated with visual processing (Kanwisher and Yovel, 2006; Booth et al., 2005; Matthews et al., 2005), with the cuneus also playing a role in response inhibition (Haldane et al., 2008). This may reflect crossmodal incongruence rather than response inhibition, as participants viewed the stimuli passively and responded only afterward, suggesting suppression of one modality over the other during incongruent trials. Another study indicated that the precuneus, along with the superior parietal lobule, is significantly activated during incongruent face processing (Hassel et al., 2020). It is also shown that the precuneus plays a key role in the prefrontal-parietal circuit during inhibitory tasks (Garavan et al., 2002; Mehren et al., 2019). The literature suggests that emotional incongruency engages the frontoparietal network in line with our findings in parietal regions like the precuneus, though further research is needed to confirm this.

Furthermore, for the contrast of emotional incongruency, we found significant activations in the Caudate and Superior Frontal Sulcus, implicated in selective inhibition, cognitive control, and emotional regulation (Schmidt et al., 2020). The caudate is crucial for controlling response interference and maintaining emotional incongruency, as it resolves response conflicts and inhibits interfering tendencies (Schmidt et al., 2020). This is relevant to our study, where emotional incongruency arises from conflicting emotions elicited by paintings and music. In Klasen et al. (2011), researchers used computer-generated avatars displaying neutral, angry, or happy facial expressions paired with disyllabic pseudowords spoken in matching or mismatching emotional prosody, with incongruent stimuli featuring conflicting facial and vocal emotions. Their fMRI results showed that incongruent stimuli engaged a frontoparietal network and the bilateral caudate nucleus, indicating a greater processing load. It also reflects the emotional conflict between the mismatched stimuli, also aligning with the results in the context of emotional conflict and monitoring, as discussed by Etkin et al., 2006 and Ochsner et al., 2009. These fMRI studies indicate the caudate’s role in managing conflicting emotional stimuli within the frontostriatal circuitry (Müller et al., 2011; Schmidt et al., 2020), which is in line with our finding for emotional incongruency.

### 4.4 Controlling low-level features (color and brightness): Original versus Fourier scrambled

Our findings comparing original paintings to Fourier Scrambled versions reveal higher-level visual and memory activations in areas like the LOC, OFC, Thalamus, and Hippocampus. This can be ascribed to the semantic context present in original paintings, which might elicit strong emotional responses and influence neural activation patterns (Doehrmann and Naumer, 2008). Additionally, given the nature of the stimuli, original paintings prompt more detailed processing and object recognition, leading to higher activation in areas like the LOC and Lingual Gyrus. The presence of semantic information and repetition of stimuli during the experiment might allow for greater association with semantic memory, potentially related to activation in the hippocampus. The contrast between Original and Fourier Scrambled paintings also reveals sensory and frontal activations in regions associated with lower-level processing of features like color and brightness, aligning with existing literature on activation patterns related to various low-level features (Cichy et al., 2011; Baldauf and Desimone, 2014; Mueller et al., 2019; Binder et al., 2017).

### 4.5 Interaction effect: emotional congruency versus incongruency when low-level features are controlled

Fourier Scrambled paintings served as an effective control condition for emotional responses by removing semantic content while retaining low-level features like color and brightness. This approach is important, as color and brightness can independently elicit emotions (Koelsch et al., 2006) and thereby confound results. Therefore, by using Fourier Scrambled stimuli and further contrasting them with original stimuli, we isolated the effects of semantic information on emotional congruency in paired paintings and music. This reduced potential confounds from crossmodal associations based on shared low-level features, such as music-color associations.

In examining the interaction effect of higher-level semantic congruency, where the difference between congruent and incongruent conditions is controlled for low-level features, we found striking activations in the ventral stream of the occipital cortex. This included visual processing areas such as the LOC, Fusiform Cortex, Lingual Gyrus in both the occipital and temporal lobes, as well as the Inferior Temporal Gyrus. These activations likely occur because congruent visual and auditory information leads to coherent emotional experiences, directing more attentional resources toward the paintings and facilitating detailed processing. Conversely, regarding emotional incongruency, studies showed that participants may automatically or attentively decrease visual processing to minimize interference during incongruent audiovisual speech streams (Deneve and Pouget, 2004; Ernst and Bülthoff, 2004). This might lead to higher activation in object and face recognition areas, reflecting the diverse objects, scenes, and faces in the paintings. These findings align with our results, where emotional congruency elicited enhanced activation along the ventral stream, while emotional incongruency led to selective inhibition, particularly in the caudate.

For the interaction contrast, we also observed activation in emotion-related regions, including the right putamen, pallidum, and amygdala, which may have been influenced by our use of emotionally rich artistic stimuli. Unlike general valence images, art can evoke stronger emotions (Tan, 2000; Silvia, 2005). We chose ‘happy’ and ‘sad’ music-painting pairs to span a broad valence range. While behavioral responses cannot confirm emotional intensity, this neural engagement supports the role of such stimuli in crossmodal emotional processing. Regarding our findings in the ventral stream and emotion-processing regions, one possible interpretation involves a neurobiological model. It suggests the swift processing of emotional signals by linking prefrontal cortex structures associated with emotions to areas responsible for object processing in the ventral stream (Rudrauf et al., 2008). This model consists of two pathways: one cortical, facilitating conscious processing of emotional stimuli, and another subcortical, involving the thalamus and amygdala, possibly supporting subconscious processing (Rudrauf et al., 2008; Garrido et al., 2012). The activation patterns in the ventral stream in our results highlight the progression from visual domains to emotion-related regions, demonstrating how coherent emotional experiences enhance processing in both visual and emotional areas (De Borst and De Gelder, 2016) The meta-analysis from Gao et al. (2019), encompassing 306 participants across 18 experiments, identified five key brain regions linked to audiovisual affective integration, including the right pSTG/STC, left aSTG/STS, right amygdala, left thalamus, and right thalamus. The regions we identified are aligned with this finding on congruent versus incongruent conditions, reflecting audiovisual affective integration.

### 4.6 Audiovisual processing and integration: superior temporal gyrus

The Crossmodal versus Unimodal contrast revealed bilateral activation in auditory regions, including Heschl’s Gyrus (HG), Planum Polare (PP) which is located in the posterior part of the Superior Temporal Gyrus (pSTG), and Planum Temporale (PT) (in the anterior part of STG), consistent with increased auditory processing for our crossmodal stimuli (Angulo-Perkins et al., 2014; Moerel et al., 2014; Trébuchon et al., 2021; Ahveninen et al., 2013). While these areas are primarily linked to auditory perception, some studies suggest that posterior STG (pSTG) may also contribute to audiovisual integration (Beauchamp et al., 2004; Hein and Knight, 2008; Obleser et al., 2006). The peak activation at pSTG was observed for Crossmodal versus Unimodal trials (MNI: 66, −18, 10; Z = 6.62). Additionally, the Congruent versus Incongruent crossmodal contrast showed bilateral activation, including in pSTG (MNI: 52, −24, 4; Z = 6.31), suggesting a potential role in the audiovisual congruency processing because both conditions involved audiovisual input. Yet, pSTG activation was stronger for congruent compared to incongruent trials. This could suggest that the effect might be specifically driven by audiovisual integration rather than auditory processing alone. Furthermore, we used the coordinate of the peak pSTG activation (MNI: 52, −24, 4; *Z* = 6.31) at Neurosynth (Poldrack et al., 2011) to check the association maps for different functional terms. Neurosynth reports a higher posterior probability for “audiovisual” tasks (0.89) than for “auditory” (0.82) or “speech perception” (0.62). An audiovisual interpretation aligns with meta-analytic findings (Gao et al., 2020), suggesting especially pSTG involvement in audiovisual tasks, particularly in emotional contexts, leading to affective audiovisual integration. However, we would like to reiterate that reverse inference (Poldrack et al., 2011) limits definitive conclusions about auditory vs. audiovisual processing.

In addition to these auditory regions, significant activation was also observed in the Parietal Operculum Cortex (POC) and the Insula. This finding aligns with the role of the parietal operculum (PO) – a segment of the central operculum – in the emotional processing of music, as it works cooperatively with the insula (Chen et al., 1995; Gebauer et al., 2014). Supporting the findings regarding the posterior operculum (PO) and insula, earlier research showed that a patient with a lesion in the central operculum and insula exhibited no emotional response to music (Griffiths et al., 2004). Furthermore, studies show that PO activation is expected during music tasks (Tanaka and Kirino, 2018), such as singing (Kleber et al., 2007) or listening to pleasant music (Koelsch et al., 2006). This suggests that parietal operculum activation is an expected response when engaging with music, whether through listening or performance, which is in line with our findings in the posterior operculum. Additionally, the insula plays a particularly important role in audiovisual integration, which is in line with our findings. A recent meta-analysis from (Gao et al., 2019) revealed that the insula shows activation for auditory attention but not visual attention. This result from Gao et al. is in line with our finding that insula exhibited increased activation when comparing crossmodal (auditory + visual) conditions to unimodal (visual-only) conditions, indicating that the addition of music enhances the processing of visual stimuli. This difference may be explained by the insula’s role in salience processing, as it is a key node in the ‘salience network’ responsible for detecting behaviorally relevant signals (Menon and Uddin, 2010; Uddin, 2015). In this study, coactivation patterns showed interactions of the amygdala and insula with STG/STS during emotional processing (Lindquist et al., 2012). This fits with our findings that span very similar regions, including the posterior operculum, insula, and superior temporal gyrus; audiovisual affective processing might require bringing together cognitive and emotion processing.

### 4.7 Limitations and future directions

In our univariate fMRI analysis, we implemented multiple comparison corrections, such as the Family-Wise Error (FWE) correction, for the broader contrast between crossmodal and unimodal comparisons. For our main contrast of Crossmodal Original versus Unimodal Original, we applied FWE and reported these results. We had very few trials in the emotional congruency and incongruency conditions, which is why we did not apply family-wise error (FWE) correction for multiple voxel comparisons, understanding that this could affect the robustness of the findings (we did include FDR-corrected findings in the tables). While we are eager to share our findings with the community, it is important to note that no multiple comparison corrections have been applied to the more specific contrasts that are zooming in on the emotional congruency effects.

Another consideration is the repetitiveness of the stimuli and the length of the experiment, which may have influenced participant engagement. However, the stimuli were carefully piloted and selected from the highest-rated happy and sad paintings and music (Wintermans, 2019), which likely enhanced emotional resonance and engagement. Although participants in the current fMRI experiment may not have perceived the stimuli as strongly congruent or incongruent, we addressed this by also collecting behavioral data. Future studies could further refine this process by expanding the stimulus set and including a broader range of emotions beyond happiness and sadness. Furthermore, in a future study, also the Fourier Scrambled versions of audio stimuli could also be used. Given that some individuals exhibit auditory dominance, future research could benefit from degrading both auditory and visual signals to further explore multisensory integration dynamics. Future research could also benefit from more dynamic presentations, which may enhance ecological validity and engagement. While our crossmodal and unimodal comparisons indicated activity in the bilateral superior temporal gyrus (STG), further examination of multivariate voxel patterns could reveal specific regions within the STG that contribute to different trial conditions, particularly in crossmodal situations.

## 5 Conclusion

In conclusion, our study explored the impact of modality on beauty ratings in both crossmodal and unimodal contexts, putting light on this aspect within the existing literature. Further, we examined emotional congruency using pairs of happy/sad paintings and music as naturalistic stimuli, combining in-scanner beauty ratings with fMRI analysis. Our findings revealed that emotional congruency elicits more robust behavioral and neural responses compared to incongruency. Specifically, congruent stimuli elicited heightened activations across brain regions involved in multisensory processing and integration, and emotional and cognitive processing. Particularly, the ventral stream activation showed the impact of semantic content when low-level features are controlled. In conclusion, this study contributes to understanding the neural mechanisms underlying audiovisual affective processing.

## Data Availability

The fMRI data supporting the findings of this study are available in the Radboud University data repository at https://doi.org/10.34973/f0xr-f791. The behavioral data and analysis scripts are available at https://doi.org/10.34973/pppa-3j30.

## References

[ref1] Ahveninen J. Huang S. Nummenmaa A. Belliveau J. W. Hung A.-Y. Jääskeläinen I. P.. (2013). Evidence for distinct human auditory cortex regions for sound location versus identity processing. Nat. Commun. 4:2585. doi: 10.1038/ncomms3585, PMID: 24121634 PMC3932554

[ref2] Albertazzi L. Canal L. Micciolo R. Hachen I. (2020). Cross-modal perceptual Organization in Works of art. I-Perception 11:204166952095075. doi: 10.1177/2041669520950750, PMID: 32922715 PMC7459189

[ref3] Angulo-Perkins A. Aubé W. Peretz I. Barrios F. A. Armony J. L. Concha L. (2014). Music listening engages specific cortical regions within the temporal lobes: differences between musicians and non-musicians. Cortex 59, 126–137. doi: 10.1016/j.cortex.2014.07.013, PMID: 25173956

[ref4] Ashburner J. Barnes G. Chen C. C. Daunizeau J. Flandin G. Friston K.. (2014). SPM12 manual. London, UK: Wellcome Trust Centre for Neuroimaging.

[ref5] Augustin M. D. Carbon C. C. Wagemans J. (2012). Artful terms: a study on aesthetic word usage for visual art versus film and music. i-Perception 3, 319–337. doi: 10.1068/i0511aap, PMID: 23145287 PMC3485829

[ref6] Baldauf D. Desimone R. (2014). Neural mechanisms of object-based attention. Science 344, 424–427. doi: 10.1126/science.1247003, PMID: 24763592

[ref8] Baumgartner T. Lutz K. Schmidt C. F. Jäncke L. (2006). The emotional power of music: how music enhances the feeling of affective pictures. Brain Res. 1075, 151–164. doi: 10.1016/j.brainres.2005.12.065, PMID: 16458860

[ref9] Beauchamp M. S. Argall B. D. Bodurka J. Duyn J. H. Martin A. (2004). Unraveling multisensory integration: patchy organization within human STS multisensory cortex. Nat. Neurosci. 7, 1190–1192. doi: 10.1038/nn1333, PMID: 15475952

[ref9001] Belardinelli M. O. Sestieri C. Di Matteo R. Delogu F. Del Gratta C. Ferretti A.. (2004). Audio-visual crossmodal interactions in environmental perception: An fMRI investigation. Cogn Process. 5, 167–174. doi: 10.1007/s10339-004-0024-0

[ref10] Binder M. Gociewicz K. Windey B. Koculak M. Finc K. Nikadon J.. (2017). The levels of perceptual processing and the neural correlates of increasing subjective visibility. Conscious. Cogn. 55, 106–125. doi: 10.1016/j.concog.2017.07.010, PMID: 28823896

[ref11] Booth J. R. Burman D. D. Meyer J. R. Lei Z. Trommer B. L. Davenport N. D.. (2005). Larger deficits in brain networks for response inhibition than for visual selective attention in attention deficit hyperactivity disorder (ADHD). J. Child Psychol. Psychiatry 46, 94–111. doi: 10.1111/j.1469-7610.2004.00337.x, PMID: 15660647

[ref12] Chen C. Y. Zimmerman R. A. Faro S. Parrish B. Wang Z. Bilaniuk L. T.. (1995). MR of the cerebral operculum: topographic identification and measurement of interopercular distances in healthy infants and children. AJNR Am. J. Neuroradiol. 16, 1677–1687. 7502974 PMC8337780

[ref13] Christensen J. F. Gaigg S. B. Gomila A. Oke P. Calvo-Merino B. (2014). Enhancing emotional experiences to dance through music: the role of valence and arousal in the cross-modal bias. Front. Hum. Neurosci. 8:757. doi: 10.3389/fnhum.2014.00757, PMID: 25339880 PMC4186320

[ref14] Cichy R. M. Chen Y. Haynes J. D. (2011). Encoding the identity and location of objects in human LOC. NeuroImage 54, 2297–2307. doi: 10.1016/j.neuroimage.2010.09.044, PMID: 20869451

[ref15] De Borst A. W. De Gelder B. (2016). Clear signals or mixed messages: inter-individual emotion congruency modulates brain activity underlying affective body perception. Soc. Cogn. Affect. Neurosci. 11, 1299–1309. doi: 10.1093/scan/nsw039, PMID: 27025242 PMC4967801

[ref16] De Gelder B. Vroomen J. (2000). The perception of emotions by ear and by eye. Cogn. Emot. 14, 289–311. doi: 10.1080/026999300378824

[ref17] Deneve S. Pouget A. (2004). Bayesian multisensory integration and cross-modal spatial links. J Physiol Paris 98, 249–258. doi: 10.1016/j.jphysparis.2004.03.011, PMID: 15477036

[ref19] Di X. Biswal B. B. (2023). A functional MRI pre-processing and quality control protocol based on statistical parametric mapping (SPM) and MATLAB. Front. Neuroimage 1:1070151. doi: 10.3389/fnimg.2022.1070151, PMID: 37555150 PMC10406300

[ref20] Doehrmann O. Naumer M. J. (2008). Semantics and the multisensory brain: how meaning modulates processes of audio-visual integration. Brain Res. 1242, 136–150. doi: 10.1016/j.brainres.2008.03.071, PMID: 18479672

[ref21] Dolan R. J. Morris J. S. De Gelder B. (2001). Crossmodal binding of fear in voice and face. Proc. Natl. Acad. Sci. 98, 10006–10010. doi: 10.1073/pnas.171288598, PMID: 11493699 PMC55568

[ref22] Eickhoff S. B. Jbabdi S. Caspers S. Laird A. R. Fox P. T. Zilles K.. (2010). Anatomical and functional connectivity of cytoarchitectonic areas within the human parietal operculum. J. Neurosci. 30, 6409–6421. doi: 10.1523/JNEUROSCI.5664-09.2010, PMID: 20445067 PMC4791040

[ref23] Ernst M. O. Bülthoff H. H. (2004). Merging the senses into a robust percept. Trends Cogn. Sci. 8, 162–169. doi: 10.1016/j.tics.2004.02.002, PMID: 15050512

[ref24] Etkin A. Egner T. Peraza D. M. Kandel E. R. Hirsch J. (2006). Resolving emotional conflict: a role for the rostral anterior cingulate cortex in modulating activity in the amygdala. Neuron 51, 871–882. doi: 10.1016/j.neuron.2006.07.029, PMID: 16982430

[ref25] Fink L. Fiehn H. Wald-Fuhrmann M. (2024). The role of audiovisual congruence in aesthetic appreciation of contemporary music and visual art. Sci. Rep. 14:20923. doi: 10.1038/s41598-024-71399-y, PMID: 39251764 PMC11384752

[ref27] Gao C. Green J. J. Yang X. Oh S. Kim J. Shinkareva S. V. (2023). Audiovisual integration in the human brain: a coordinate-based meta-analysis. Cereb. Cortex 33, 5574–5584. doi: 10.1093/cercor/bhac443, PMID: 36336347 PMC10152097

[ref28] Gao C. Weber C. E. Shinkareva S. V. (2019). The brain basis of audiovisual affective processing: evidence from a coordinate-based activation likelihood estimation meta-analysis. Cortex 120, 66–77. doi: 10.1016/j.cortex.2019.05.016, PMID: 31255920

[ref29] Gao C. Weber C. E. Wedell D. H. Shinkareva S. V. (2020). An fMRI study of affective congruence across visual and auditory modalities. J. Cogn. Neurosci. 32, 1251–1262. doi: 10.1162/jocn_a_01553, PMID: 32108554

[ref30] Gao C. Wedell D. H. Green J. J. Jia X. Mao X. Guo C.. (2018). Temporal dynamics of audiovisual affective processing. Biol. Psychol. 139, 59–72. doi: 10.1016/j.biopsycho.2018.10.001, PMID: 30291876

[ref31] Garavan H. Ross T. J. Murphy K. Roche R. A. Stein E. A. (2002). Dissociable executive functions in the dynamic control of behavior: inhibition, error detection, and correction. NeuroImage 17, 1820–1829. doi: 10.1006/nimg.2002.1326, PMID: 12498755

[ref32] Garrido M. I. Barnes G. R. Sahani M. Dolan R. J. (2012). Functional evidence for a dual route to the amygdala. Curr. Biol. 22, 129–134. doi: 10.1016/j.cub.2011.11.056 22209532 PMC3267035

[ref33] Gebauer L. Skewes J. Westphael G. Heaton P. Vuust P. (2014). Intact brain processing of musical emotions in autism spectrum disorder, but more cognitive load and arousal in happy vs. sad music. Front. Neurosci. 8:192. doi: 10.3389/fnins.2014.00192, PMID: 25076869 PMC4098021

[ref34] Gerdes A. Alpers G. W. Braun H. Köhler S. Nowak U. Treiber L. (2021). Emotional sounds guide visual attention to emotional pictures: an eye-tracking study with audio-visual stimuli. Emotion 21:679. doi: 10.1037/emo0000729, PMID: 32191086

[ref36] Giannos K. Athanasopoulos G. Cambouropoulos E. (2021). Cross-modal associations between harmonic dissonance and visual roughness. Music. Sci. 4:20592043211055484. doi: 10.1177/20592043211055484

[ref37] Griffiths T. D. Warren J. D. Dean J. L. Howard D. (2004). “When the feeling’s gone”: a selective loss of musical emotion. J. Neurol. Neurosurg. Psychiatry 75, 344–345. doi: 10.1136/jnnp.2003.015586 14742630 PMC1738902

[ref38] Haldane M. Cunningham G. Androutsos C. Frangou S. (2008). Structural brain correlates of response inhibition in bipolar disorder I. J. Psychopharmacol. 22, 138–143. doi: 10.1177/0269881107082955, PMID: 18308812

[ref39] Hassel S. Sharma G. B. Alders G. L. Davis A. D. Arnott S. R. Frey B. N.. (2020). Reliability of a functional magnetic resonance imaging task of emotional conflict in healthy participants. Hum. Brain Mapp. 41, 1400–1415. doi: 10.1002/hbm.24883, PMID: 31794150 PMC7267954

[ref40] Hein G. Doehrmann O. Müller N. G. Kaiser J. Muckli L. Naumer M. J. (2007). Object familiarity and semantic congruency modulate responses in cortical audiovisual integration areas. J. Neurosci. 27, 7881–7887. doi: 10.1523/JNEUROSCI.1740-07.2007, PMID: 17652579 PMC6672730

[ref41] Hein G. Knight R. T. (2008). Superior temporal sulcus—it's my area: or is it? J. Cogn. Neurosci. 20, 2125–2136. doi: 10.1162/jocn.2008.20148, PMID: 18457502

[ref44] Isaacson A. Assis A. Adi-Japha E. (2023). “Listening” to paintings: synergetic effect of a cross-modal experience on subjective perception. Empir. Stud. Arts 41, 433–464. doi: 10.1177/02762374231155742

[ref45] Jansma H. Roebroeck A. Münte T. F. (2014). A network analysis of audiovisual affective speech perception. Neuroscience 256, 230–241. doi: 10.1016/j.neuroscience.2013.10.047, PMID: 24184115

[ref46] Jeong J.-W. Diwadkar V. A. Chugani C. D. Sinsoongsud P. Muzik O. Behen M. E.. (2011). Congruence of happy and sad emotions in music and faces modifies cortical audiovisual activation. Neuro Image 54, 2973–2982. doi: 10.1016/j.neuroimage.2010.11.017, PMID: 21073970

[ref47] Kanwisher N. Yovel G. (2006). The fusiform face area: a cortical region specialized for the perception of faces. Philos. Trans. R. Soc. B Biol. Sci. 361, 2109–2128. doi: 10.1098/rstb.2006.1934, PMID: 17118927 PMC1857737

[ref48] Klasen M. Kenworthy C. A. Mathiak K. A. Kircher T. T. J. Mathiak K. (2011). Supramodal representation of emotions. J. Neurosci. 31, 13635–13643. doi: 10.1523/JNEUROSCI.2833-11.2011, PMID: 21940454 PMC6623280

[ref49] Kleber B. Birbaumer N. Veit R. Trevorrow T. Lotze M. (2007). Overt and imagined singing of an Italian aria. NeuroImage 36, 889–900. doi: 10.1016/j.neuroimage.2007.02.053, PMID: 17478107

[ref50] Koelsch S. Fritz T. Cramon V. D. Y. Müller K. Friederici A. D. (2006). Investigating emotion with music: an fMRI study. Hum. Brain Mapp. 27, 239–250. doi: 10.1002/hbm.20180, PMID: 16078183 PMC6871371

[ref51] Kravitz D. J. Saleem K. S. Baker C. I. Ungerleider L. G. Mishkin M. (2013). The ventral visual pathway: an expanded neural framework for the processing of object quality. Trends Cogn. Sci. 17, 26–49. doi: 10.1016/j.tics.2012.10.011, PMID: 23265839 PMC3532569

[ref52] Kreifelts B. Ethofer T. Grodd W. Erb M. Wildgruber D. (2007). Audiovisual integration of emotional signals in voice and face: an event-related fMRI study. NeuroImage 37, 1445–1456. doi: 10.1016/j.neuroimage.2007.06.020, PMID: 17659885

[ref53] Kreifelts B. Ethofer T. Huberle E. Grodd W. Wildgruber D. (2010). Association of trait emotional intelligence and individual fMRI-activation patterns during the perception of social signals from voice and face. Hum. Brain Mapp. 31, 979–991. doi: 10.1002/hbm.20913, PMID: 19937724 PMC6871025

[ref54] Kreifelts B. Ethofer T. Shiozawa T. Grodd W. Wildgruber D. (2009). Cerebral representation of non-verbal emotional perception: fMRI reveals audiovisual integration area between voice-and face-sensitive regions in the superior temporal sulcus. Neuropsychologia 47, 3059–3066. doi: 10.1016/j.neuropsychologia.2009.07.001, PMID: 19596021

[ref55] Law E. Von Ahn L. (2009). Input-agreement: a new mechanism for collecting data using human computation games. In Proceedings of the SIGCHI conference on human factors in computing systems (1197–1206).

[ref56] Law E. L. Von Ahn L. Dannenberg R. B. Crawford M. (2007). Tag ATune: a game for music and sound annotation: ISMIR.

[ref57] Levitan C. A. Charney S. A. Schloss K. B. Palmer S. E. (2015). The smell of jazz: crossmodal correspondences between music, odor, and emotion. CogSci Proceedings of the Annual Meeting of the Cognitive Science Society, 37, 1326–1331. Available at: https://escholarship.org/uc/item/1hb8c91r

[ref58] Lindquist K. A. Wager T. D. Kober H. Bliss-Moreau E. Barrett L. F. (2012). The brain basis of emotion: a meta-analytic review. Behav. Brain Sci. 35, 121–143. doi: 10.1017/S0140525X11000446, PMID: 22617651 PMC4329228

[ref59] Matthews S. C. Simmons A. N. Arce E. Paulus M. P. (2005). Dissociation of inhibition from error processing using a parametric inhibitory task during functional magnetic resonance imaging. Neuroreport 16, 755–760. doi: 10.1097/00001756-200505120-00020, PMID: 15858420

[ref60] Mehren A. Özyurt J. Thiel C. M. Brandes M. Lam A. P. Philipsen A. (2019). Effects of acute aerobic exercise on response inhibition in adult patients with ADHD. Sci. Rep. 9:19884. doi: 10.1038/s41598-019-56332-y, PMID: 31882652 PMC6934617

[ref61] Menon V. Uddin L. Q. (2010). Saliency, switching, attention, and control: a network model of insula function. Brain Struct. Funct. 214, 655–667. doi: 10.1007/s00429-010-0262-0, PMID: 20512370 PMC2899886

[ref63] Moerel M. De Martino F. Formisano E. (2014). An anatomical and functional topography of human auditory cortical areas. Front. Neurosci. 8:225. doi: 10.3389/fnins.2014.00225, PMID: 25120426 PMC4114190

[ref64] Mok P. P. Li G. Li J. J. Ng H. T. Cheung H. (2019). Cross-modal association between vowels and colours: a cross-linguistic perspective. J. Acoust. Soc. Am. 145, 2265–2276. doi: 10.1121/1.5096632, PMID: 31046303

[ref65] Molholm S. Ritter W. Javitt D. C. Foxe J. J. (2004). Multisensory visual–auditory object recognition in humans: a high-density electrical mapping study. Cereb. Cortex 14, 452–465. doi: 10.1093/cercor/bhh007, PMID: 15028649

[ref66] Motoki K. Saito T. Nouchi R. Sugiura M. (2020). Cross-modal correspondences between temperature and taste attributes. Front. Psychol. 11:571852. doi: 10.3389/fpsyg.2020.571852, PMID: 33101140 PMC7546214

[ref67] Mueller S. De Haas B. Metzger A. Drewing K. Fiehler K. (2019). Neural correlates of top-down modulation of haptic shape versus roughness perception. Hum. Brain Mapp. 40, 5172–5184. doi: 10.1002/hbm.24764, PMID: 31430005 PMC6864886

[ref69] Müller V. I. Habel U. Derntl B. Schneider F. Zilles K. Turetsky B. I.. (2011). Incongruence effects in crossmodal emotional integration. Neuro Image 54, 2257–2266. doi: 10.1016/j.neuroimage.2010.10.047, PMID: 20974266 PMC8007888

[ref70] Obleser J. Boecker H. Drzezga A. Haslinger B. Hennenlotter A. Roettinger M.. (2006). Vowel sound extraction in anterior superior temporal cortex. Hum. Brain Mapp. 27, 562–571. doi: 10.1002/hbm.20201, PMID: 16281283 PMC6871493

[ref71] Ochsner K. N. Hughes B. Robertson E. R. Cooper J. C. Gabrieli J. D. (2009). Neural systems supporting the control of affective and cognitive conflicts. J. Cogn. Neurosci. 21, 1841–1854. doi: 10.1162/jocn.2009.21129 PMC6558970 18823233

[ref72] Palmer S. E. Schloss K. B. Xu Z. Prado-León L. R. (2013). Music–color associations are mediated by emotion. Proc. Natl. Acad. Sci. 110, 8836–8841. doi: 10.1073/pnas.1212562110, PMID: 23671106 PMC3670360

[ref73] Parise C. V. Spence C. (2009). ‘When birds of a feather flock together’: synesthetic correspondences modulate audiovisual integration in non-synesthetes. PLoS One 4:e5664. doi: 10.1371/journal.pone.0005664, PMID: 19471644 PMC2680950

[ref74] Parise C. Spence C. (2013). “Audiovisual cross-modal correspondences in the general population” in The Oxford handbook of synesthesia. eds. Simner J. Hubbard E. M. (Oxford: Oxford University Press), 790–815.

[ref75] Pehrs C. Zaki J. Schlochtermeier L. H. Jacobs A. M. Kuchinke L. Koelsch S. (2015). The temporal pole top-down modulates the ventral visual stream during social cognition. Cereb. Cortex 27, bhv226–bhv792. doi: 10.1093/cercor/bhv226, PMID: 26604273

[ref76] Peirce J. W. (2007). Psycho Py—psychophysics software in Python. J. Neurosci. Methods 162, 8–13. doi: 10.1016/j.jneumeth.2006.11.017, PMID: 17254636 PMC2018741

[ref77] Peirce J. W. (2009). Generating stimuli for neuroscience using PsychoPy. Front. Neuroinform. 2:10. doi: 10.3389/neuro.11.010.2008 19198666 PMC2636899

[ref78] Pelowski M. (2017). Move me, astonish me… delight my eyes and brain: the Vienna integrated model of top-down and bottom-up processes in art perception (VIMAP) and corresponding affective, evaluative, and neurophysiological correlates. Phys. Life Rev. 21, 80–125. doi: 10.1016/j.plrev.2017.02.003, PMID: 28347673

[ref79] Petrini K. Crabbe F. Sheridan C. Pollick F. E. (2011). The music of your emotions: neural substrates involved in detection of emotional correspondence between auditory and visual music actions. PLoS One 6:e19165. doi: 10.1371/journal.pone.0019165, PMID: 21559468 PMC3084768

[ref80] Poldrack R. A. Mumford J. A. Nichols T. E. (2011). Handbook of functional MRI data analysis. 1st Edn. Cambridge: Cambridge University Press.

[ref81] Public Catalogue Foundation (n.d.) Art UK: welcome to the nation’s art [Art Database] Available online at: https://artuk.org (Accessed March 01, 2019).

[ref82] Rančić K. Marković S. (2019). The perceptual and aesthetic aspects of the music-paintings congruence. Vision 3:65. doi: 10.3390/vision3040065, PMID: 31756887 PMC6969919

[ref84] Robins D. L. Hunyadi E. Schultz R. T. (2009). Superior temporal activation in response to dynamic audio-visual emotional cues. Brain Cogn. 69, 269–278. doi: 10.1016/j.bandc.2008.08.007, PMID: 18809234 PMC2677198

[ref85] Rosenfeld N. Steffens J. (2019). Effects of audiovisual congruency on perceived emotions in film. Psychomusicol. Music Mind Brain 29, 200–208. doi: 10.1037/pmu0000242

[ref86] Rudrauf D. David O. Lachaux J. P. Kovach C. K. Martinerie J. Renault B.. (2008). Rapid interactions between the ventral visual stream and emotion-related structures rely on a two-pathway architecture. J. Neurosci. 28, 2793–2803. doi: 10.1523/JNEUROSCI.3476-07.2008, PMID: 18337409 PMC6670659

[ref87] Saluja S. Stevenson R. J. (2018). Cross-modal associations between real tastes and colors. Chem. Senses 43, 475–480. doi: 10.1093/chemse/bjy033, PMID: 29868904

[ref88] Schmidt C. C. Timpert D. C. Arend I. Vossel S. Fink G. R. Henik A.. (2020). Control of response interference: caudate nucleus contributes to selective inhibition. Sci. Rep. 10:20977. doi: 10.1038/s41598-020-77744-1, PMID: 33262369 PMC7708449

[ref89] Seeley W. P. (2012). Hearing how smooth it looks: selective attention and crossmodal perception in the arts. Essays Philos. 13, 498–517. doi: 10.7710/1526-0569.1434

[ref90] Seijdel N. Schoffelen J. M. Hagoort P. Drijvers L. (2024). Attention drives visual processing and audiovisual integration during multimodal communication. J. Neurosci. 44:e0870232023. doi: 10.1523/JNEUROSCI.0870-23.2023, PMID: 38199864 PMC10919203

[ref91] Sepulcre J. Sabuncu M. R. Yeo T. B. Liu H. Johnson K. A. (2012). Stepwise connectivity of the modal cortex reveals the multimodal organization of the human brain. J. Neurosci. 32, 10649–10661. doi: 10.1523/JNEUROSCI.0759-12.2012, PMID: 22855814 PMC3483645

[ref92] Silvia P. J. (2005). Emotional responses to art: from collation and arousal to cognition and emotion. Rev. Gen. Psychol. 9, 342–357. doi: 10.1037/1089-2680.9.4.342

[ref93] Spence C. (2011). Crossmodal correspondences: a tutorial review. Atten. Percept. Psychophys. 73, 971–995. doi: 10.3758/s13414-010-0073-7, PMID: 21264748

[ref94] Spence C. (2020). Assessing the role of emotional mediation in explaining crossmodal correspondences involving musical stimuli. Multisens. Res. 33, 1–29. doi: 10.1163/22134808-20191469, PMID: 31648195

[ref95] Spence C. Deroy O. (2013). How automatic are crossmodal correspondences? Conscious. Cogn. 22, 245–260. doi: 10.1016/j.concog.2012.12.006, PMID: 23370382

[ref97] Talsma D. Senkowski D. Soto-Faraco S. Woldorff M. G. (2010). The multifaceted interplay between attention and multisensory integration. Trends Cogn. Sci. 14, 400–410. doi: 10.1016/j.tics.2010.06.008, PMID: 20675182 PMC3306770

[ref98] Tan E. S. (2000). “Emotion, art, and the humanities” in Handbook of emotions. eds. Lewis M. Haviland J. M. (New York: Guilford Press).

[ref99] Tanaka S. Kirino E. (2018). The parietal opercular auditory-sensorimotor network in musicians: a resting-state fMRI study. Brain Cogn. 120, 43–47. doi: 10.1016/j.bandc.2017.11.001, PMID: 29122368

[ref100] The MathWorks Inc. (2021) MATLAB version: 9.11.0 (R2021b), Natick, Massachusetts: The MathWorks Inc. Available online at: https://www.mathworks.com

[ref101] Trébuchon A. Alario F. X. Liégeois-Chauvel C. (2021). Functional topography of auditory areas derived from the combination of electrophysiological recordings and cortical electrical stimulation. Front. Hum. Neurosci. 15:702773. doi: 10.3389/fnhum.2021.702773, PMID: 34489664 PMC8418073

[ref102] Uddin L. Q. (2015). Salience processing and insular cortical function and dysfunction. Nat. Rev. Neurosci. 16, 55–61. doi: 10.1038/nrn3857, PMID: 25406711

[ref103] Van Lier R. Koning A. (2017). Listening to paintings [Poster presentation]. The Visual Science of Art Conference (VSAC), Berlin, Germany, 5:395.

[ref104] Vuilleumier P. (2005). How brains beware: neural mechanisms of emotional attention. Trends Cogn. Sci. 9, 585–594. doi: 10.1016/j.tics.2005.10.011, PMID: 16289871

[ref105] Wang X. Guo X. Chen L. Liu Y. Goldberg M. E. Xu H. (2017). Auditory to visual cross-modal adaptation for emotion: psychophysical and neural correlates. Cereb. Cortex 27, 1337–1346. doi: 10.1093/cercor/bhv321, PMID: 26733537 PMC6074898

[ref106] Wang Q. J. Wang S. Spence C. (2016). “Turn up the taste”: assessing the role of taste intensity and emotion in mediating Crossmodal correspondences between basic tastes and pitch. Chem. Senses 41, 345–356. doi: 10.1093/chemse/bjw007, PMID: 26873934 PMC4840871

[ref108] WikiArt (n.d.) Wikiart visual encyclopedia [Art database]. Available online at: https://www.wikiart.org (Accessed March 01, 2019).

[ref109] Wintermans A. (2019). Crossmodal art perception: congruency versus incongruency. Nijmegen, Netherlands: Radboud University.

