# Supplementary material for: An fMRI study of crossmodal emotional congruency and the role of semantic content in the aesthetic appreciation of naturalistic art
Source: Front Neurosci. 2025 Jul 30;19:1516070. doi: 10.3389/fnins.2025.1516070 (PMC12345300; doi:10.3389/fnins.2025.1516070)
Supplement: Supplementary file 1 [file Data_Sheet_1.pdf]

Supplementary Material

1 Confirmatory Analysis

1.1: Effects of Modality and Semantics

In this section, we analyze univariate fMRI data focusing on two main contrasts: the effect of Modality, which compares Crossmodal versus Unimodal stimuli, and the effect of Semantics, which distinguishes between Original and Fourier Scrambled stimuli. These two analyses serve as a basic confirmation to ensure the validity of our experimental design.

1.1.1 Modality: CrossModal > Unimodal

This fMRI contrast explores differences in brain activations between Crossmodal trials—encompassing Congruent Original, Incongruent Original, Congruent Fourier Scrambled, and Incongruent Fourier Scrambled conditions—and Unimodal trials, which are comprised of either Original or Fourier Scrambled presentations. Figure 1 reveals several brain regions with stronger activations for Crossmodal trials compared to Unimodal within the auditory cortices. These include the Planum Polare and the anterior Superior Temporal Gyrus, both primarily linked to auditory processing, and Heschl's Gyrus, a key auditory region. Additionally, the Planum Temporale and the posterior Superior Temporal Gyrus, associated mainly with audiovisual integration, show significant activations (Beauchamp et al., 2004; Hein & Knight, 2008; Obleser et al., 2006) (Figure 1a). Furthermore, the Parietal Operculum Cortex and the Insula, which are known to facilitate connections between auditory, parietal, and frontal areas, also exhibited notable activations. This is supported by functional connectivity analyses (Sepulcre et al., 2012; Eickhoff et al., 2010) (Figure 1b). Our hypothesis posited that Crossmodal stimuli would induce stronger responses in sensory cortices and audiovisual integration areas. The outcomes corroborate this, showing activations consistent with our expectations for Crossmodal trials. The detailed results of the CrossModal > Unimodal fMRI contrast for specified brain regions and their coordinates (Supplementary Table 1).

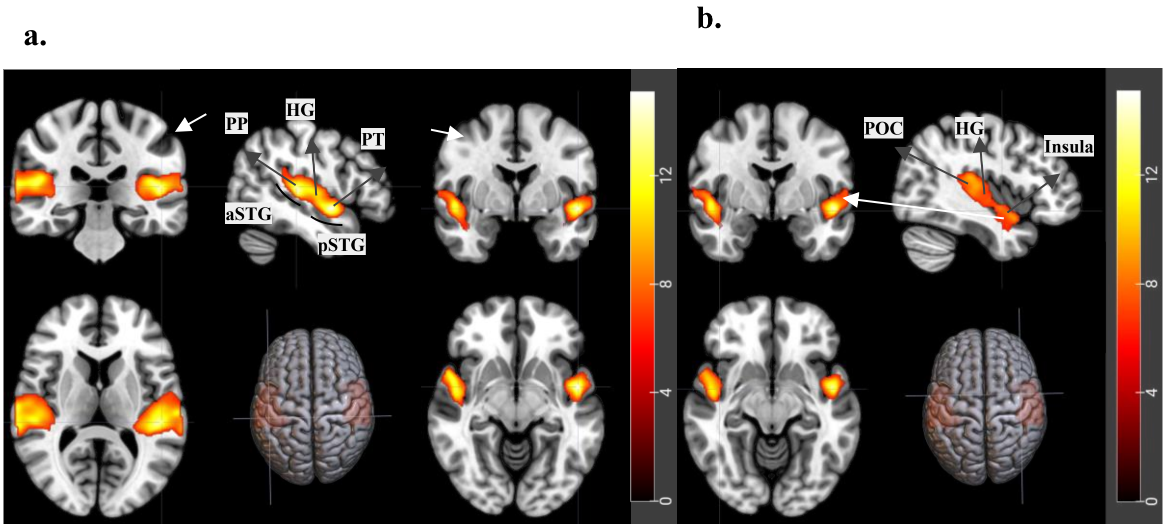

Supplementary Figure 1. **fMRI results for the Crossmodal > Unimodal contrast.** t-statistics for 2nd level analysis, N=21, FWEcorr < 0.05, and cluster size>20. **(A)** Key auditory regions such as the Planum Polare (PP), Heschl's Gyrus (HG), and Planum Temporale (PT) spanning at anterior (aSTG) and posterior (pSTG) segments of the Superior Temporal Gyrus are highlighted. **(B)** Features activations in the Parietal Operculum Cortex (POC), Heschl's Gyrus (HG), and the Insula.

1.1.2 Semantics: Original > Fourier Scrambled

The contrast of Semantics revealed higher activation in the inferior Lateral Occipital Cortex, extending to the Occipital Fusiform Cortex (Figure 2a, b, c). Notably, the Lateral Occipital Cortex is recognized for its role in object recognition. At the same time, the Occipital Fusiform Cortex is associated with face recognition (affective processing is primarily driven by face processing (Kanwisher & Yovel, 2006). Additionally, substantial activations were observed in the bilateral Thalamus and Hippocampus (Figure 2d). The analysis also highlighted significant activations in the Lingual Gyrus, Cuneus, and Precuneus (Figure 2e), which are associated with higher-level visual processing. The detailed results of the Original > Fourier Scrambled fMRI contrast for specified brain regions and their coordinates (Suuplementary, Table 1).

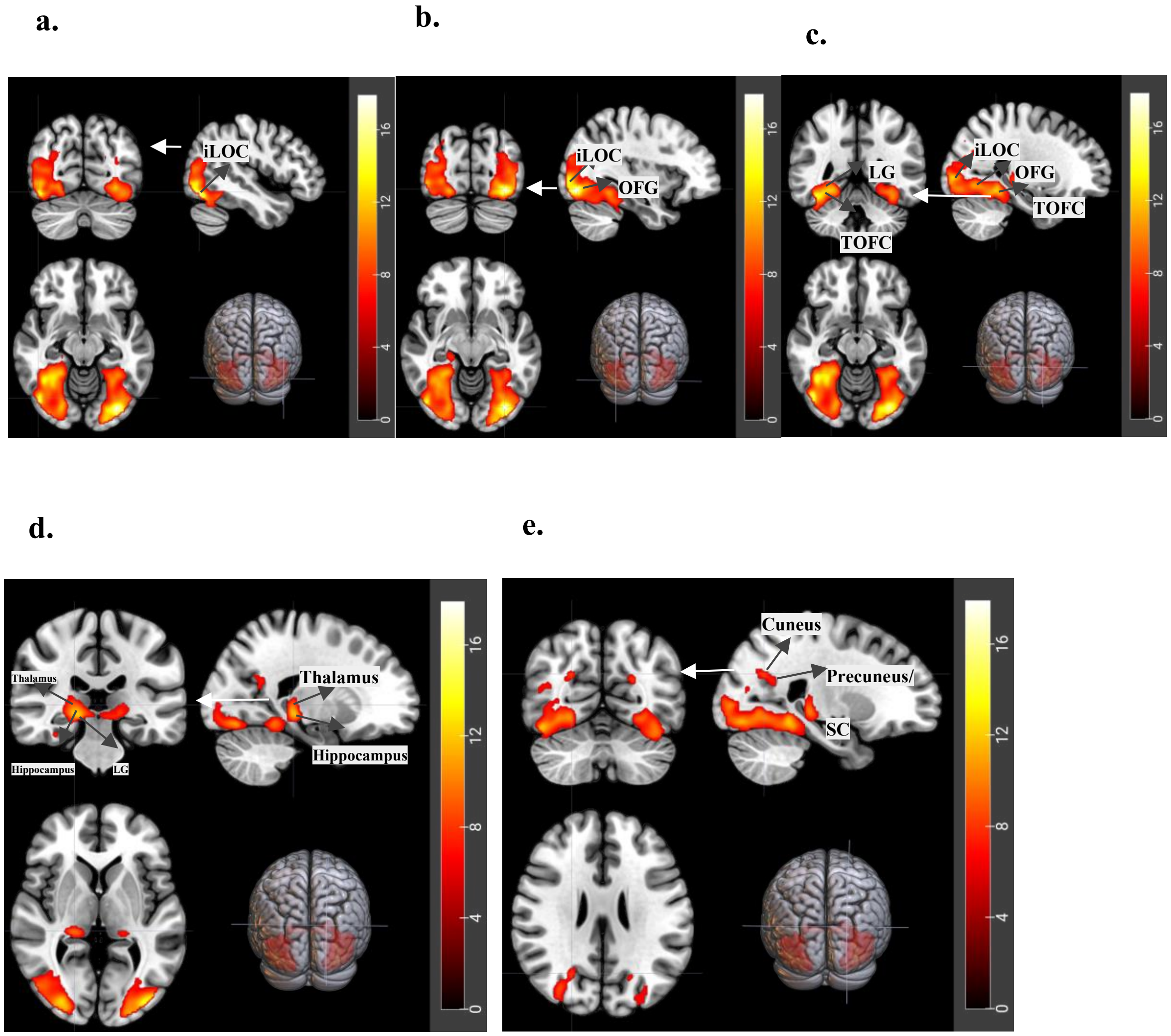

Supplementary Figure 2. **fMRI results for Original > Fourier Scrambled contrast.** t-statistics for 2nd level analysis, N=21 subjects, with a threshold of FWE < 0.05 and cluster size>20. **a.** Displays key visual processing area, the inferior Lateral Occipital Cortex (iLOC) across sagittal, axial, and coronal planes. b. Features activations in the iLOC and Occipital Fusiform Gyrus (OFG). **c.** Focuses on the ventral stream for the high visual processing areas the temporal Occipital Fusiform Cortex, Lingual Gyrus (LG), OFG, and iLOC. **d.** Reveals activity in the Thalamus, Hippocampus, and LG. **e.** Highlights the Cuneus, Precuneus, and Supracalcarine Cortex.

Supplementary Table 1: Cluster Characteristics and Coordinates for Modality and Semantics of fMRI Univariate Analysis

		Peak Coordinates (MNI)			
Region	*Cluster Size*	*x (mm)*	*y(mm)*	*z(mm)*	*Z Score*
**Crossmodal > Unimodal (*FWE<.05*)**					
Planum Temporale/ Parietal Operculum Cortex / Posterior STG	2832	50	-28	14	6.61
Planum Polare/ Central Opercular Cortex		50	2	-6	6.58
Planum Temporale/ Posterior STG		66	-18	10	6.55
Planum Polare/ Anterior STG	2613	-50	-2	-8	6.97
Planum Temporale/ Heschl's Gyrus		-50	-28	6	6.79

Planum Temporale/ Posterior STG		-62	-22	8	6.35

Original > Fourier Scrambled (*FWE<.05*)

Lateral Occipital Cortex - inferior	5169	46	-70	-14	7.23
Temporal Occipital Fusiform Cortex		40	-52	-16	7.08
Temporal Occipital Fusiform Cortex/ Lingual Gyrus		30	-52	-14	6.76
Occipital Fusiform Gyrus/ Lateral Occipital Cortex - inferior	3787	-34	-80	-10	7.38
Lateral Occipital Cortex - inferior		-38	-88	0	7.30
Occipital Fusiform Gyrus		-24	-84	-14	6.83
Left Thalamus / Left Hippocampus	143	-18	-32	-2	5.52
		-10	-32	-6	5.20

Cuneal Cortex / Precuneus Cortex	42	-22	-66	24	5.54

Supplementary Table 2 shows the brain activations for specific regions and corresponding clusters and coordinates for Congruency Fourier Scrambled versus Incongruency Fourier Scrambled.

Supplementary Table 2: Cluster Characteristics and Coordinates for Congruency Fourier Scrambled versus Incongruency Fourier Scrambled

		Peak Coordinates (MNI)			
Region	*Cluster Size*	*x (mm)*	*y(mm)*	*z(mm)*	*Z Score*
**Congruency Fourier Scrambled > Incongruency Fourier Scrambled (*p <.001*)**					
Heschl's Gyrus	1655	52	-20	8	6,26
		56	-34	12	4,27
		64	-36	14	4,00
Planum Temporale	1596	-52	-26	8	5,52
		-54	-6	4	4,41
Occipital Pole/ Lingual Gyrus	1305	-14	-100	2	4,94

		14	-90	-6	4,88
		14	-100	12	4,65
Precentral Gyrus/ Superior Frontal Gyrus	680	-30	-14	60	4,37
		-32	-24	48	3,64
		-48	-20	52	3,30
Juxtapositional Lobule Cortex/ Precentral Gyrus	397	-8	-10	52	4,30
		-6	0	64	3,75
		-6	6	44	3,58
Postcentral Gyrus/ Supramarginal Gyrus	233	52	-20	48	3,49
		34	-30	48	3,45
		46	-26	50	3,41
Juxtapositional Lobule Cortex/ Precentral Gyrus	101	10	2	62	4,00

1.1.3 Main Contrast for Congruency (Original + Fourier Scrambled) versus Incongruency (Original + Fourier Scrambled)

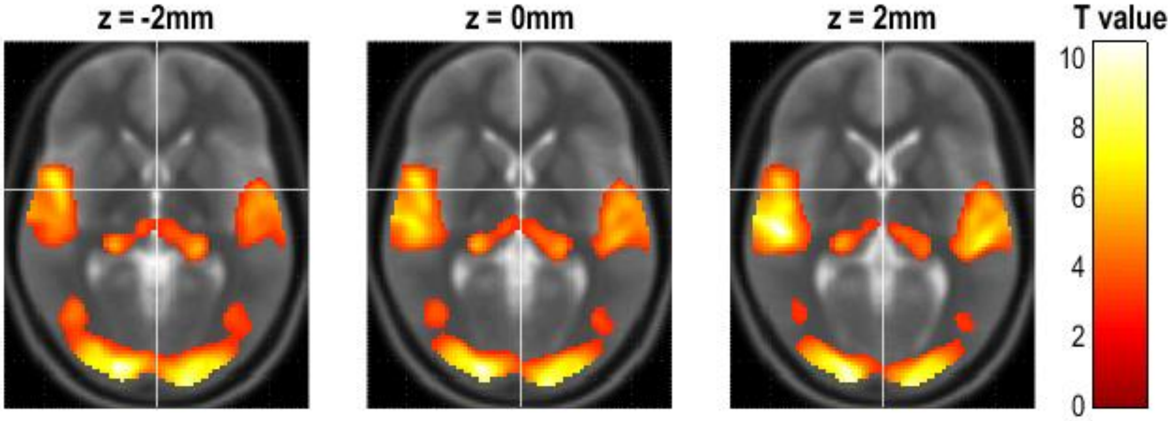

Supplementary Figure 3: fMRI results for Congruency (Original+Fourier Scrambled) versus Incongruency (Original+Fourier Scrambled) main contrast. t-statistics for 2nd level analysis, N=21 subjects, with a threshold of FDR< 0.05

Supplementary Figure 3 demonstrates regions almost identical to Congruency Original versus Incongruency Original. The activated areas in this contrasts are visual areas such as the Lateral Occipital Cortex, Occipital Pole, and auditory areas Heschl's Gyrus, Planum Temporal.

Supplementary Table 3: Cluster Characteristics and Coordinates for Congruency (Original+Fourier Scrambled) versus Incongruency (Original+Fourier Scrambled)

		Peak Coordinates (MNI)			
Region	*Cluster Size*	*x (mm)*	*y(mm)*	*z(mm)*	*Z Score*
**Congruency Original > Incongruency Original (FDR,** *p <.005***)**					
Occipital Pole/ Lingual Gyrus	7607	16	-90	-6	6.02
Temporal Pole/Planum Polare, Heschl's Gyrus/ Planum Temporale, Superior Temporal Gyrus – posterior	5817	50	-24	-8	7.0

Juxtapositional Lobule Cortex/ Precentral Gyrus	4769	-8	0	54	4.61
Planum Temporale/ Heschl's Gyrus	3441	52	-26	8	5.62
Right Thalamus	720	22	-28	0	4.35
Left Thalamus/ Hippocampus	720	22	-28	0	4.35

2. Beauty Ratings for Happy/Sad Pairs in Emotional (in)congruency

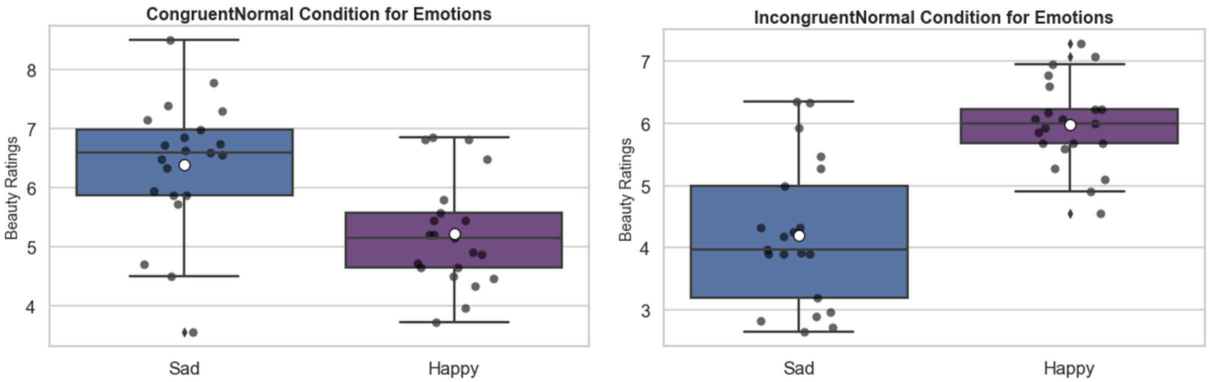

Supplementary Figure 4: *A*. Beauty ratings for Congruent pairs (Sad-Sad vs. Happy-Happy) in Painting-Music combinations, showing a significant difference ($p < 0.0001$). *B*. Beauty ratings for Incongruent pairs, where Sad paintings were paired with Happy music and Happy paintings with Sad music
